# The multidrug ABC transporter BmrC*/*BmrD of *Bacillus subtilis* is regulated via a ribosome-mediated transcriptional attenuation mechanism

**DOI:** 10.1093/nar/gku832

**Published:** 2014-09-12

**Authors:** Ewoud Reilman, Ruben A. T. Mars, Jan Maarten van Dijl, Emma L. Denham

**Affiliations:** Department of Medical Microbiology, University Medical Center Groningen, University of Groningen, Hanzeplein 1, P.O. box 30001, 9700 RB Groningen, the Netherlands

## Abstract

Expression of particular drug transporters in response to antibiotic pressure is a critical element in the development of bacterial multidrug resistance, and represents a serious concern for human health. To obtain a better understanding of underlying regulatory mechanisms, we have dissected the transcriptional activation of the ATP-binding cassette (ABC) transporter BmrC/BmrD of the Gram-positive model bacterium *Bacillus subtilis*. By using promoter-GFP fusions and live cell array technology, we demonstrate a temporally controlled transcriptional activation of the *bmrCD* genes in response to antibiotics that target protein synthesis. Intriguingly, *bmrCD* expression only occurs during the late-exponential and stationary growth stages, irrespective of the timing of the antibiotic challenge. We show that this is due to tight transcriptional control by the transition state regulator AbrB. Moreover, our results show that the *bmrCD* genes are co-transcribed with *bmrB* (*yheJ*), a small open reading frame immediately upstream of *bmrC* that harbors three alternative stem-loop structures. These stem-loops are apparently crucial for antibiotic-induced *bmrCD* transcription. Importantly, the antibiotic-induced *bmrCD* expression requires translation of *bmrB*, which implies that BmrB serves as a regulatory leader peptide. Altogether, we demonstrate for the first time that a ribosome-mediated transcriptional attenuation mechanism can control the expression of a multidrug ABC transporter.

## INTRODUCTION

Since the clinical introduction of antibiotics, bacteria have evolved efficient mechanisms to counteract their effects. This has resulted in the emergence of multidrug resistant (MDR) bacterial pathogens, which is a serious threat to public health as the treatment of infections caused by these pathogens is becoming increasingly difficult (1). An effective strategy for bacteria to achieve resistance to a multitude of antibiotics is the expression of efflux pumps, such as ATP-binding cassette (ABC) transporters, that can actively excrete harmful substances from the cell (1,2).

The model bacterium *Bacillus subtilis*, a close relative of notoriously drug-resistant Gram-positive bacteria, such as *Staphylococcus aureus*, is equipped with an extensive repertoire of genes that encode for potential drug efflux transporters (3). These include a significant number of ABC transporters (4). Eight of the 78 ABC transport systems that are encoded by the genome of *B. subtilis* have been classified as potential MDR-like transporters (4). Among these eight is the heterodimeric ABC transporter YheI/YheH, which was renamed BmrC/BmrD (in short BmrCD) due to its homology to the LmrC/LmrD ABC transporter of *Lactococcus lactis* (5).

BmrCD was the first multidrug ABC transporter in *B. subtilis* that was shown to function as a heterodimer. Torres *et al.* (6) used inside-out membrane vesicles to demonstrate that BmrCD can mediate efflux of several fluorescent substrates of MDR-ABC transporters, including Hoechst 33342, doxorubicin and mitoxantrone. In addition, they investigated the transcriptional response of *B. subtilis* to subinhibitory concentrations of 46 different antibiotics. Eleven of these antibiotics, most of which act as inhibitors of protein synthesis, were shown to significantly increase the transcription of the *bmrCD* operon (6). These included ribosome-targeted antibiotics, such as chloramphenicol, erythromycin and gentamycin, which was consistent with an earlier study on antibiotic-induced gene expression in *B. subtilis* (7).

In the present study, we have dissected the transcriptional regulation of the *bmrCD* genes in response to ribosome-targeted antibiotics to obtain a better understanding of the underlying regulatory mechanisms. The results show that induction of *bmrCD* is controlled at two levels. First, this process is tightly restricted to the transition and stationary growth phases by the main transition state regulator AbrB. Second, we show that antibiotic-induced expression of *bmrCD* is regulated via a dedicated ribosome-mediated transcriptional attenuation mechanism that requires the *yheJ-*encoded leader peptide. In view of the location of *yheJ* immediately upstream of *bmrC*, and the intimate relationship between *yheJ* translation and the expression of *bmrCD*, we have renamed *yheJ* to *bmrB*.

## MATERIALS AND METHODS

### Plasmids, bacterial strains and growth condition

The plasmids and bacterial strains used in this study are listed in Supplementary Table S1, and the primers used for plasmid constructions in Supplementary Table S2. *B. subtilis* 168 and *Escherichia coli* TG1 were grown with vigorous agitation in Lysogeny broth (LB; Difco laboratories) at 37°C. Where appropriate, the growth medium was supplemented with antibiotics: ampicillin 100 μg/ml, spectinomycin 100 μg/ml, chloramphenicol 5 μg/ml (*B. subtilis*) or 10 μg/ml (*E. coli*).

### Construction of chromosomally integrated transcriptional 5′-*gfp* fusions

Transcriptional green fluorescent protein (GFP) fusions were constructed using the integrative pBaSysBioII plasmid, as described previously (8). The 5′ upstream regions of *bmrB* or *bmrC* were amplified by polymerase chain reaction (PCR) with the Phusion Hot Start High-Fidelity DNA Polymerase (Finnzymes, NEB), and then joined with the pBaSysBioII plasmid by Ligation-Independent Cloning (LIC). The resulting plasmids were used to transform *B. subtilis* 168 as described by Kunst and Rapoport (9). Since pBaSysBioII cannot replicate in *B. subtilis*, its derivatives carrying the cloned 5′ upstream regions of *bmrB* or *bmrC* integrated at the respective loci via single cross-over recombination. This placed the respective *gfp* fusion in single copy on the chromosome at the original locus and, at the same time the sequence cloned in pBaSysBioII was duplicated (for details see 8). For each transcriptional-GFP fusion, three independent clones were selected and used for further experiments. In addition, genomic DNA isolated from one of these clones was used as a template to PCR-amplify the complete or a truncated version of the respective transcriptional GFP fusion. The resulting PCR-amplified fragments were cloned into pRMC, a LIC-adapted derivative of the pXTC plasmid (10) that allows the incorporation of genes via double cross-over recombination into the chromosomal *amyE* gene. Importantly, the xylose-inducible promoter of pXTC was removed from pRMC, making this plasmid suitable for driving GFP expression exclusively from inserted promoter and regulatory sequences. Integration of the different transcriptional GFP fusions into the *amyE* locus was confirmed by growing transformants on starch-containing plates and testing the absence of α-amylase secretion by staining of the plates with iodine as described previously (10).

### Construction of plasmid-borne transcriptional *bmrB-gfp* fusions

Plasmid pRM3 was constructed by combining the backbone of plasmid pHB201 (11) with the xylose-inducible promoter from pXTC (10) using circular polymerase extension cloning and primers listed in Supplementary Table S2 (12). In this manner, the *Asc*I LIC site was introduced into pRM3 by primer overlap. Two different fragments of *bmrB* including the 5′ untranslated region (UTR) and coding sequence, either with or without the terminator region, were amplified by PCR. The resulting DNA fragments were fused by overlap-PCR with *gfpmut3*, including an optimal ribosome-binding site (RBS) (8). The resulting overlapped PCR products were gel-extracted and cloned into plasmid pRM3 via LIC (13) resulting in plasmids pRM3-*bmrB*162 and pRM3-*bmrB*109. Mutations in pRM3-*bmrB*ΔTerm were introduced by site-directed mutagenesis using the *bmrB-gfp* fusion of pRM3-*bmrB*162 as a template. For this purpose, *bmrB-gfp* was amplified by PCR in two separate fragments, one of which included the required mutations. Subsequently, these PCR fragments were joined by overlap-PCR, gel-extracted and cloned into plasmid pRM3 via LIC. The sequences required for pRM3-*bmrB*Δstart and pRM3-*bmrB*−CodonOpt, were commercially synthesized by Integrated DNA Technologies (http://eu.idtdna.com/). PCR fragments of these synthesized sequences, consisting of the front part of *bmrB*, were fused to the terminator region and GFP by overlap-PCR.

### Live cell array (LCA) analyses

LCA analyses with *B. subtilis* were performed as described previously (8,14,15). All strains used for LCA analysis were grown overnight in LB, diluted 1000-fold and grown in 100 μl cultures in 96-well flat bottom microtiter plates (Greiner Bio-One). Cultures were grown for 14 h in a Biotek synergy 2 plate reader, monitoring both growth by optical density readings at 600 nm (OD_600nm_) and GFP fluorescence (excitation 485/20 nm, emission 528/20 nm) at 5- or 10-min intervals. The promoter-GFP strains were either exposed to subinhibitory concentrations of antibiotics or mock-treated during early exponential growth (OD_600 nm_ corrected for 1 cm path length = ∼0.5). Background fluorescence from the isogenic wild-type control strain not expressing GFP was subtracted. Arbitrary transcriptional activity units (TAU) represent the increase in GFP expression levels during each 5- or 10-min interval and were calculated using the equation: (GFP*^t^* − GFP^*t*−1^)/OD_600_*^t^* (where *t* represents a given time point at which fluorescence was measured, and *t* − 1 the preceding time point at which fluorescence was measured). Expression of the transcriptional-GFP fusions on pRM3 was induced in exponentially growing cells (OD_600 nm_ ∼0.18) by the addition of xylose (0.1% final concentration) either with or without supplementation of lincomycin (0.75 μg/ml). For the experiments with strains containing pRM3-borne *gfp* fusions, the final GFP activity levels were corrected by subtracting the GFP activity levels measured for non-induced cultures. The xylose-induced GFP activity levels thus obtained were expressed in arbitrary units (AU). Data depicted in the LCA figures were derived from one of three representative experiments.

### RNA extraction, reverse transcription and real-time PCR

*B. subtilis* 168 was inoculated from an overnight culture into 150 ml LB medium to an OD_600_ of 0.05. During exponential growth (OD_600_ ∼0.6) the cells were either mock-treated or exposed to lincomycin (0.5 μg/ml) for 150 min. Next, total RNA was extracted according to the method described by Eymann *et al.* (16). The RNA was reverse transcribed into cDNA using Taqman RT-PCR reagents (Applied Biosystems, LifeTechnologies) according to the manufacturer's protocol. The resulting cDNA was used as a PCR template to confirm the structure of the *bmrB/bmrC/bmrD* operon (in short *bmrBCD*) with Taq polymerase (Life Technologies) and the quantitative PCR (qPCR) primers *bmrB*-foward and *bmrC*-reverse (Supplementary Table S2). Real-time qPCR (GoTaq qPCR Master Mix, Promega) was performed according to the manufacturer's protocol using an Applied Biosystems 7500 Real-time PCR system and the primer sets specified in Supplementary Table S2. The *recF* and *ssrA* genes were included as reference genes. Data analysis was carried out using the accompanying Applied Biosystems software package (v.2.0.5).

## RESULTS

### Transition phase-dependent induction of *bmrCD* by ribosome-targeted antibiotics

**Figure 1.**
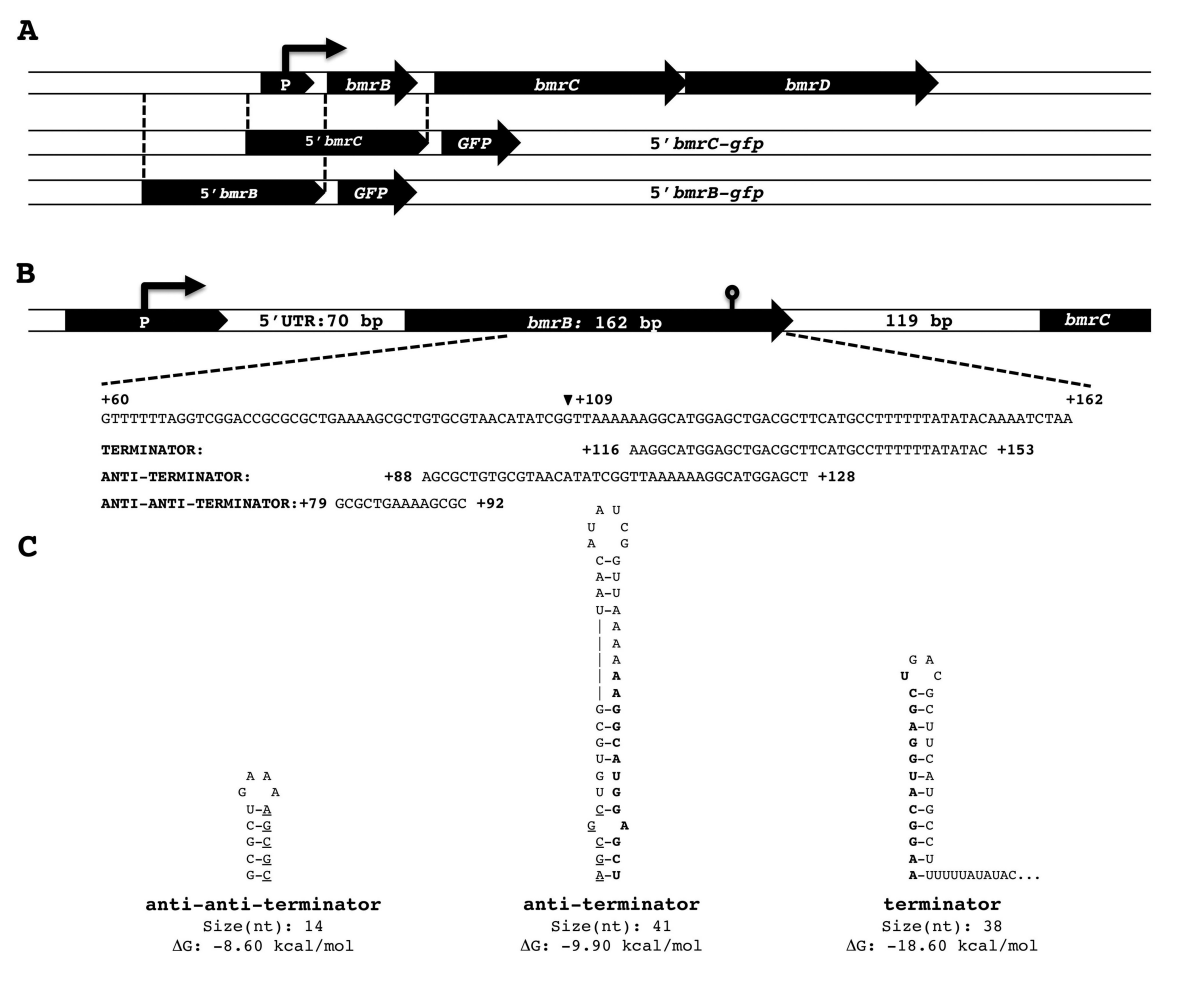
Genomic context of the *bmrCD* locus and transcriptional GFP fusions. **A.** The top line depicts the genomic context of the ABC transporter genes *bmrC* and *bmrD*, which are preceded by the small open reading frame *bmrB.* The two lines below represent the relative positions of the two regions selected for construction of the transcriptional 5′*bmrC*-*gfp* and 5′*bmrB*-*gfp* fusions. **B.** Detailed representation of the regulatory region upstream of *bmrC*, including the segment of *bmrB* (nucleotides 60–162) in which predicted terminator, anti-terminator and anti-anti-terminator structures are encoded. These three putative structures are assigned with their nucleotide positions relative to the first nucleotide of *bmrB*. The black arrowhead marks nucleotide 109 after which *bmrB* was truncated in pRM3-*bmrB*109. In addition, the 5′UTR upstream of *bmrB* and the intergenic region between *bmrB* and *bmrC* are indicated. **C.** Structures of the putative anti-anti-terminator, anti-terminator and terminator, with their respective sizes and Gibbs free energy (Δ*G*) values, as predicted by RibEx: Riboswitch Explorer. The overlap in the terminator and anti-terminator structures is marked in bold, and the overlap in the anti-terminator and anti-anti-terminator structures is marked by underlining.

The BmrCD ABC transporter was previously shown to be regulated by exposure to several ribosome-targeted antibiotics (6,7). To gain further insights into the mechanisms involved in this regulation, the genomic environment of the *bmrCD* genes was carefully mapped using data from a recent high-resolution transcriptional analysis of *B*. *subtilis* grown under 104 different conditions (17). Based on these data, which are schematically represented in Supplementary Figure S1A, we postulated that the *bmrC* and *bmrD* genes are co-transcribed with *bmrB* (previously known as *yheJ*), a small and as yet uncharacterized gene of 162 bp that ends 119 bp upstream from the start codon of *bmrC* (Figure 1A and B). This view was confirmed by a reverse transcription PCR analysis (Supplementary Figure S1B). It thus seems that the *bmrBCD* operon is transcribed from a conjoint promoter which, according to the *B. subtilis* Expression Data Browser (http://genome.jouy.inra.fr/seb), is located ∼70 bp upstream of *bmrB* (Figure 1B).

**Figure 2.**
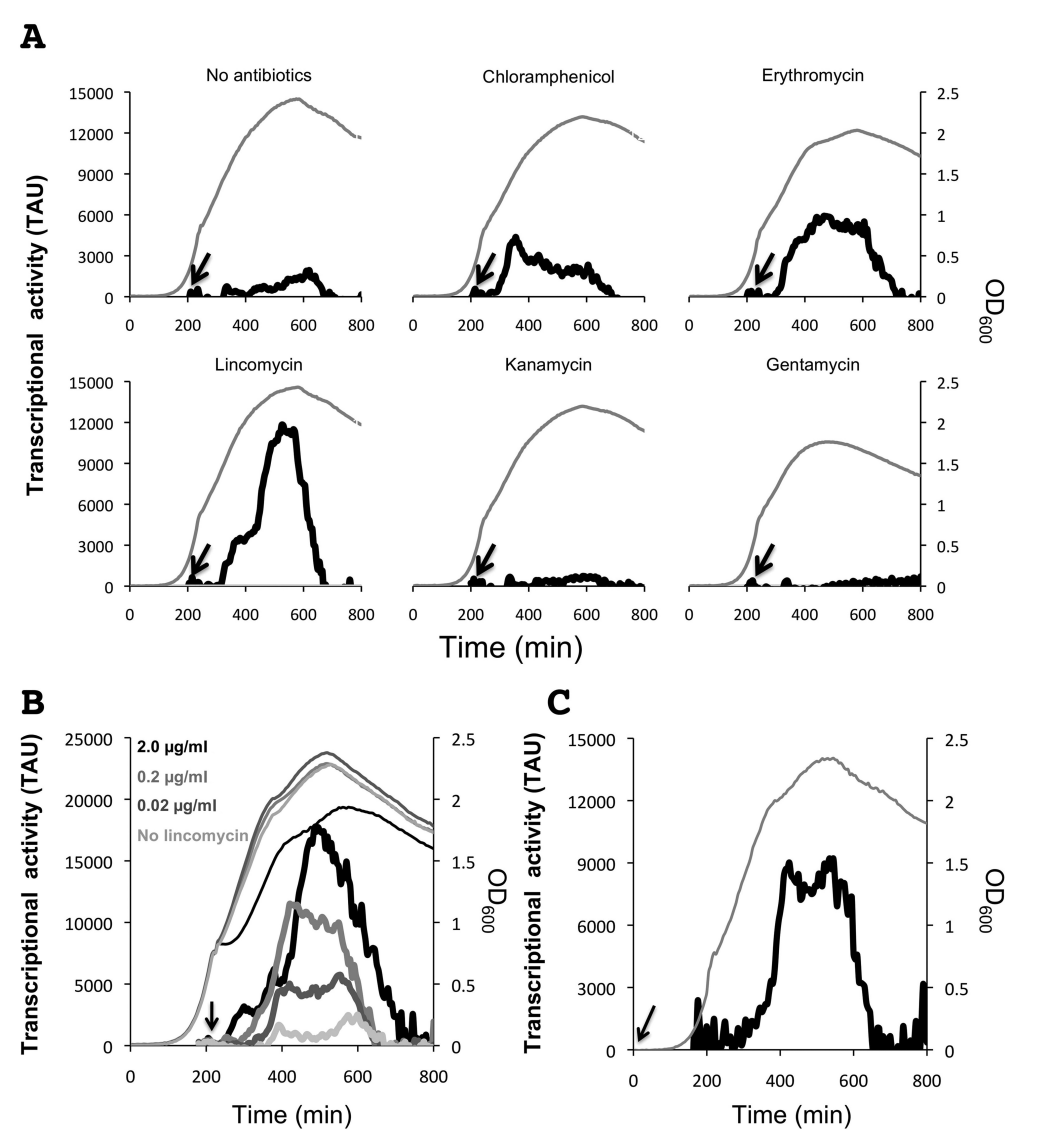
LCA real-time analysis of *bmrCD* transcription. **A.** Growth of *B. subtilis* BSBII-*5*′*bmrC*-*gfp* (OD_600 nm_; gray line) and *bmrC* transcriptional activity (solid black line) in response to the ribosome-targeted antibiotics chloramphenicol, erythromycin, lincomycin, kanamycin or gentamycin. Arrows indicate the time points at which antibiotics or an equivalent volume of water (‘no antibiotics’) were added. **B.** Dose-dependent response of 5′*bmrC*-*gfp* transcription to increasing concentrations of lincomycin. Bold lines indicate transcriptional activity and thin lines indicate bacterial growth. The arrow indicates the time point at which lincomycin was added. **C.** Transcriptional response of *B. subtilis* BSBII-5′*bmrC*-*gfp* to an early exposure to lincomycin. The bold line indicates transcriptional activity and the dashed line indicates bacterial growth. Lincomycin (final concentration: 0.02 μg/ml) was added at the start of the culture, as indicated by the arrow. Cultures entered the transition phase between exponential and post-exponential growth at OD_600_ of ∼1.

To gain further insight into the mechanisms involved in *bmrCD* expression, we transcriptionally fused the 600-bp region upstream from the −18 position of *bmrC* to *gfp* and integrated one copy of this fusion by single cross-over recombination into the native *bmrC* locus of the *B. subtilis* chromosome (Figure 1A). The resulting strain 168 BSBII-5′*bmrC-gfp* was then used to monitor the antibiotic-induced expression of *bmrCD* in ‘real-time’ following the LCA approach as detailed in the Materials and Methods. To determine the baseline *gfp* expression in *B. subtilis* 168 BSBII-5′*bmrC-gfp*, this strain was cultured in LB without antibiotics. As shown in Figure 2A, relatively little transcriptional activity (∼500 TAU) was detectable during exponential growth, but the activity increased during the late transition and early stationary growth phases to ∼1950 TAU. Next, exponentially growing cells (OD_600nm_ ∼0.5) carrying the 5′*bmrC-gfp* fusion were exposed to subinhibitory concentrations of the ribosome-targeted antibiotics chloramphenicol (0.2 μg/ml), erythromycin (0.02 μg/ml), lincomycin (0.2 μg/ml), kanamycin (0.2 μg/ml) or gentamycin (0.2 μg/ml). The addition of chloramphenicol or erythromycin resulted in a slight reduction in the growth rate and a slight drop in the maximum OD_600 nm_ that was reached (Figure 2A). Following addition of either of these two antibiotics, the transcriptional activity of the 5′*bmrC-gfp* fusion remained unaltered at baseline levels for ∼75 min and then increased during a period of ∼50–200 min to a maximum (∼4350–5900 TAU, respectively) after which it gradually returned to baseline levels. Interestingly, while the presence of 0.2 μg/ml lincomycin had no inhibitory effect on growth, it triggered the strongest induction of the 5′*bmrC-gfp* fusion (Figure 2A). After a ∼75-min delay, lincomycin induced the transcriptional activity of the 5′*bmrC-gfp* fusion to a maximum of ∼11 800 TAU and this level was sustained for ∼90 min before returning to baseline. In contrast, the antibiotics kanamycin and gentamycin did not induce the 5′*bmrC-gfp* fusion. The latter observation was unexpected as earlier studies had suggested that gentamycin would induce *bmrCD* expression (6), a finding that we were unable to reproduce. In fact, the *gfp* transcription even seemed to decrease in response to these antibiotics. In this respect it is noteworthy that the latter two antibiotics target the 30S ribosomal subunit, while the *bmrCD*-activating antibiotics chloramphenicol, erythromycin and lincomycin are specific for the 50S subunit.

We subsequently verified the dose-dependence of the response to the most potent inducer of *bmrCD* transcription, lincomycin, by subjecting *B. subtilis* 168 BSBII-5′*bmrC-gfp* to increasing concentrations of lincomycin. This showed that the transcriptional response toward lincomycin was indeed dose-dependent (Figure 2B). The lowest concentration of lincomycin, (0.02 μg/ml) induced the 5′*bmrC-gfp* fusion to a maximum of ∼5800 TAU. By increasing the lincomycin concentration 10-fold (0.2 μg/ml), the transcriptional activity was doubled, and a further 10-fold increase (2 μg/ml) induced the *bmrC* promoter activity to ∼17 800 TAU. This relatively high concentration of lincomycin slightly affected growth of the 168 BSBII-5′*bmrC-gfp* strain (Figure 2B).

Intriguingly, the transcription of *bmrCD* was highly growth phase-dependent as evidenced by the ∼75-min delay between the administration of chloramphenicol, erythromycin or lincomycin and the induction of promoter activity. Specifically, this delayed promoter activation coincided with the late transition stage in growth. To further investigate the apparent growth phase-dependent transcriptional activation of *bmrCD*, lincomycin (0.02 μg/ml) was administered to the culture at the start of the experiment (T0; Figure 2C). Also in this case, the 5′*bmrC-gfp* fusion remained largely silent until the ∼300 min time point after which its activity increased to a peak of ∼9100 TAU. The GFP activity remained constant for ∼200 min before returning to basal level. Thus, transcriptional activation of *bmrCD* followed exactly the same pattern as observed when the antibiotic was added after 200 min of growth. This shows that *bmrCD* expression as reflected by the 5′*bmrC-gfp* fusion is tightly regulated in a growth-phase-dependent manner.

To exclude the possibility that the delayed transcriptional response of the 5′*bmrC-gfp* fusion was caused by a direct effect of the antibiotics on GFP expression levels, a strain with an isopropyl β-D-1-thiogalactopyranoside (IPTG)-inducible transcriptional 5′-GFP fusion,*B. subtilis* 168 BSBII *spac-gfp*, was included in the experiments (Supplementary Figure S2). A final concentration of 0.1 mM IPTG was added to exponentially growing bacteria, which, as expected, resulted in an immediate and strong induction of the *spac-gfp* fusion. Importantly, the addition of lincomycin (2.0 μg/ml) had no detectable effect on the IPTG-induced GFP production, excluding a direct inhibitory effect of this antibiotic on the production of active GFP. Taken together, these observations imply that *bmrCD* expression is induced by a subset of ribosome-targeted antibiotics, and that this induction is limited to the late transition and early stationary growth phases.

### The *bmrBCD* promoter determines growth phase-controlled *bmrCD* expression

**Figure 3.**
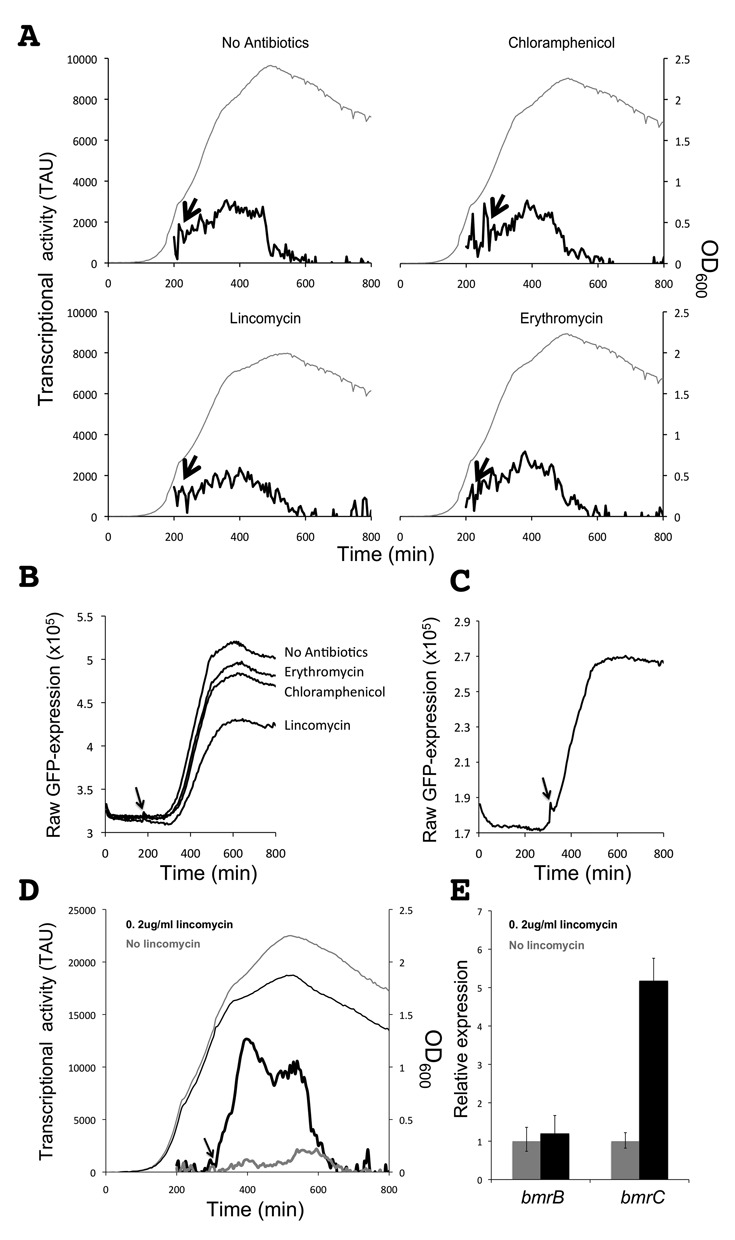
LCA real-time analysis of *bmrB* transcription. **A.** Growth of *B. subtilis* BSBII-*5*′*bmrB*-*gfp* (OD_600 nm_; gray line) and *bmrB* transcriptional activity (solid black line) in response to chloramphenicol, lincomycin or erythromycin. Arrows indicate the time points at which antibiotics or an equivalent volume of water (‘no antibiotics’) were added. Cultures entered the transition phase between exponential and post-exponential growth at OD_600_ of ∼1. **B.** Graphs from raw GFP values as recorded with the Biotek synergy 2 plate reader. These raw values were used to calculate the TAU in panel A. Note that details of the transcriptional activity profiles in panel A are partly obscured by background noise. **C** and **D.** Lincomycin-induced transcription of the 5′*bmrC*-*gfp* fusion coincides with the time point at which transcription of the *bmrB*-*gfp* fusion starts to increase. Strains carrying the 5′*bmrB*-*gfp* fusion (C) or the 5′*bmrC*-*gfp* fusion (D) were grown in parallel; lincomycin was added to the BSBII-5′*bmrC*-*gfp* strain once GFP fluorescence in the BSBII-5′*bmrB*-*gfp* strain started to increase. Note that panel C shows the raw GFP expression levels while panel D shows TAU. **E.** Quantitative real-time PCR analysis on RNA isolated from *B. subtilis* 168 during transition-phase growth, either in the presence or absence of lincomycin (0.5 μg/ml).

Notably, the 5′*bmrC-gfp* fusion was constructed to monitor *bmrCD* transcription and, for this purpose, the 600-bp region directly upstream of *bmrC* was cloned into pBaSysBioII. As a consequence, transcription of the 5′*bmrC-gfp* fusion was not only subject to control by the upstream *bmrBCD* promoter region, but also to possible regulatory sequences within *bmrB* (Figure 1A). To establish whether the antibiotic-induced and temporally controlled transcription of *bmrCD* could be entirely attributed to the *bmrBCD* promoter, a second pBaSysBioII-based GFP reporter fusion was constructed using the 600-bp region directly upstream of *bmrB* (Figure 1A). One copy of the resulting transcriptional 5′*bmrB-gfp* fusion was introduced by single cross-over recombination into the *bmrB* locus of the *B. subtilis* chromosome. Next, the resulting strain BSBII-5′*bmrB-gfp* was grown under the same antibiotic stress conditions as described above. As shown in Figure 3A and B, the 5′*bmrB-gfp* fusion displayed a growth phase-dependent expression profile, similar to the 5′*bmrC-gfp* fusion. In all tested conditions, the 5′*bmrB-gfp* fusion was activated during the transition from exponential to stationary growth (Figure 3A and B), matching the time point of the antibiotic-induced activation observed in the BSBII-5′*bmrC-gfp* strain (Figure 2). However, the BSBII-5′*bmrB-gfp* strain did not respond to the antibiotics chloramphenicol, erythromycin and lincomycin (Figure 3A), which induced the transcription of *bmrCD*. This led to the hypothesis that particular features within the upstream region of *bmrCD* could be responsible for antibiotic-induced transcription of the two ABC transporter genes. Subsequent *in silico* analysis of the region upstream of *bmrC* revealed a perfect intrinsic terminator-like structure within the coding region of *bmrB*, together with alternative anti-terminator and anti-anti-terminator structures as defined by the RibEx: Riboswitch Explorer algorithm (18) (Figure 1B and C).

Conceivably, the terminator structure within *bmrB* could play a role in the antibiotic-induced activation of the 5′*bmrC-gfp* construct while, as indicated above, expression of the complete *bmrBCD* operon is most likely directed from the promoter located upstream of *bmrB*. Expression of the *bmrCD* transporter genes, however, would rely on the decision of the RNA polymerase to either stop transcription at the terminator site or continue transcription of *bmrCD*. As a first approach to test this idea, the BSBII-5′*bmrC-gfp* and BSBII-5′*bmrB-gfp* strains were monitored in parallel and lincomycin was added to the BSBII-5′*bmrC-gfp* strain directly after the first fluorescence signal from BSBII-5′*bmrB-gfp* strain was detectable (Figure 3C). The addition of lincomycin at this point resulted in an immediate transcriptional response in the BSBII-5′*bmrC-gfp* strain (Figure 3C and D). This showed that the onset of transcription of *bmrB* coincides with the onset of antibiotic-induced transcription of *bmrCD*, and it was in agreement with our hypothesis that the putative terminator in *bmrB* controls the antibiotic-induced transcription of *bmrCD*. To provide additional evidence for lincomycin induction of the *bmrCD* genes, we isolated RNA from *B*. *subtilis* 168 cultures that were either treated with 0.5 μg/ml lincomycin or mock-treated. Real-time qPCR demonstrated that the relative expression level of *bmrB* was stable in these conditions, whereas transcription of *bmrC* was increased 5- to 7-fold in lincomycin-treated cells (Figure 3E). This was in full agreement with the data obtained from the experiments with the promoter GFP fusions. Furthermore, we verified that there is no alternative promoter within *bmrB* that controls the antibiotic-induced transcription of *bmrCD* by removing the *bmrB* promoter region from the 5′*bmrC-gfp* fusion. Both the truncated and complete 5′*bmrC-gfp* fusions were cloned into pRMC and were integrated into the *amyE* locus. The complete 5′*bmrC-gfp* fusion was inducible by lincomycin, whereas the truncated 5′*bmrC-gfp* fusion lacking the *bmrB* promoter region was not inducible and displayed only baseline levels of GFP (Supplementary Figure S3). Together these findings verified that transcription of the *bmrBCD* operon is indeed initiated from a promoter upstream of *bmrB*. This conjoint promoter is activated during the transition- and early-stationary growth stages and is not responsive to ribosome-targeted antibiotics, such as lincomycin.

### The transition state regulator AbrB represses transcription of the *bmrBCD* operon during exponential growth

**Figure 4.**
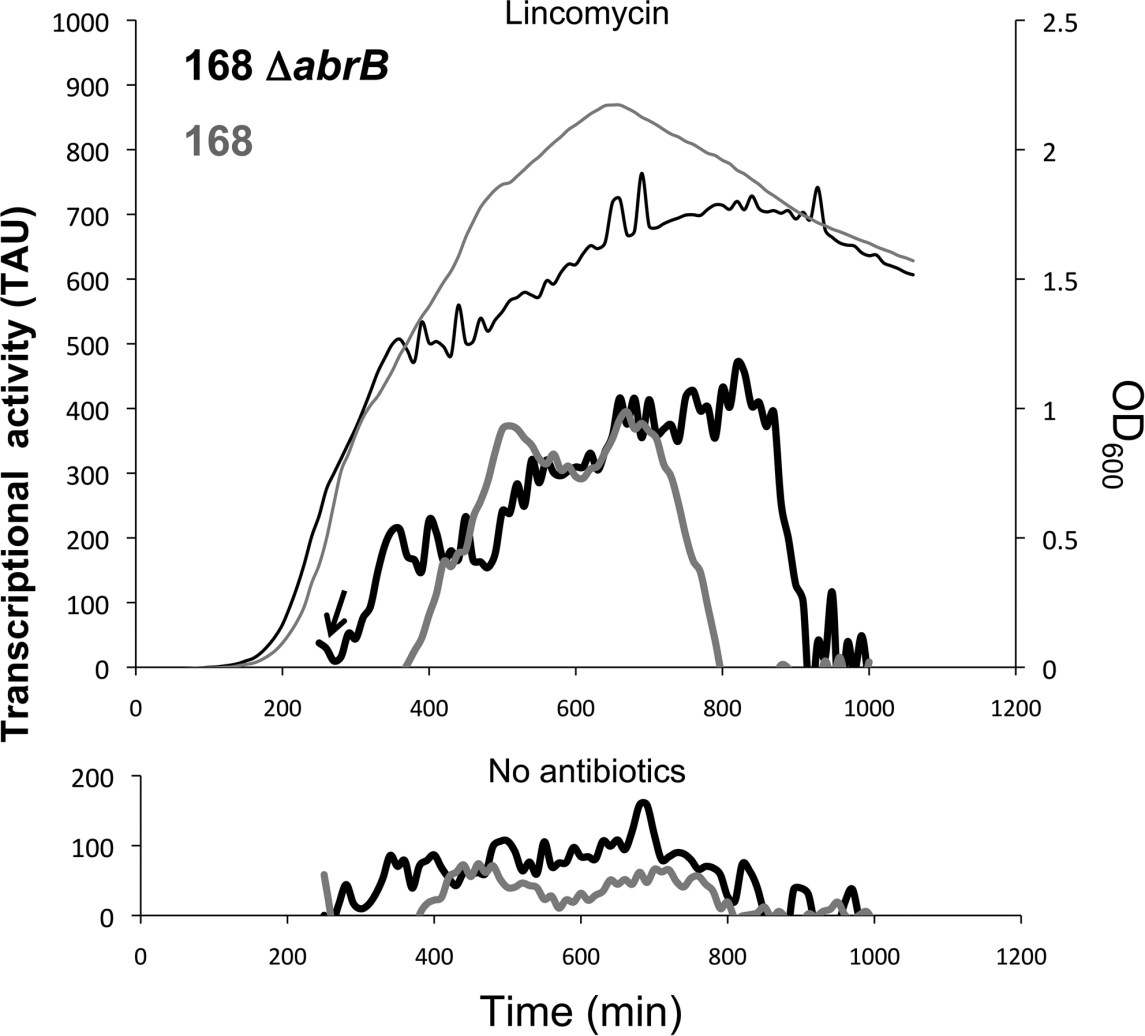
AbrB determines the growth phase-dependent transcription of *bmrCD.* The upper panel shows the transcriptional activity (TAU) of the 5′*bmrC*-*gfp* fusion in the parental strain *B. subtilis* 168 (thick gray line) and in an *abrB* deletion mutant (thick black line) upon lincomycin induction. The thin lines depict growth of the *abrB*-proficient (gray) and *abrB*-deficient (black) strains carrying the 5′*bmrC*-*gfp* fusion. Lincomycin was added to the cells during exponential growth as indicated with an arrow. The lower panel shows the transcriptional activity (TAU) of the 5′*bmrC*-*gfp* fusion in the parental strain 168 (thick gray line) and the *abrB* deletion mutant (thick black line) grown in the absence of lincomycin. Note that the patterns of the expression profiles for strain 168 are qualitatively similar to those shown in Figures 2 and 3D, but that the arbitrary TAU are quantitatively different due to the use of plate readers with different detector sensitivity.

Since expression of the *bmrBCD* operon was strictly retained to the transition and early-stationary growth stages, we investigated whether this could be attributed to the transition state regulator AbrB. Supporting this idea, a potential AbrB-binding site upstream of *bmrB* was previously identified in a genome-wide AbrB-binding study (19). In addition, Chumsakul *et al.* showed that transcription of *bmrBCD* was elevated in an *abrB* deletion mutant, where *bmrB* and *bmrCD* mRNA levels increased by 4- and 2-fold, respectively. To verify that AbrB represses the early transcriptional activation of the *bmrBCD* operon, we introduced the 5′*bmrC-gfp* fusion into a Δ*abrB* background. Indeed, the *abrB* deletion resulted in derepression, precluding the growth phase-dependent expression of the 5′*bmrC*-*gfp* fusion (Figure 4, lower panel). When exponentially growing Δ*abrB* cells with the 5′*bmrC-gfp* fusion were subjected to lincomycin (0.2 μg/ml), this resulted in a strong expression of GFP. However, in contrast to the *abrB*-proficient strain, which displayed the reported delay, the transcriptional activation of the 5′*bmrC-gfp* fusion in response to lincomycin was considerably faster in the Δ*abrB* strain (Figure 4, upper panel). Transcriptional activation occurred within 20 min after the addition of lincomycin. We therefore conclude that transcription of the *bmrBCD* operon is restrained to the transition and early-stationary growth stages via repression by the global transition-state regulator AbrB.

### The intrinsic terminator within the *bmrB*-coding region controls the expression of *bmrCD*

**Figure 5.**
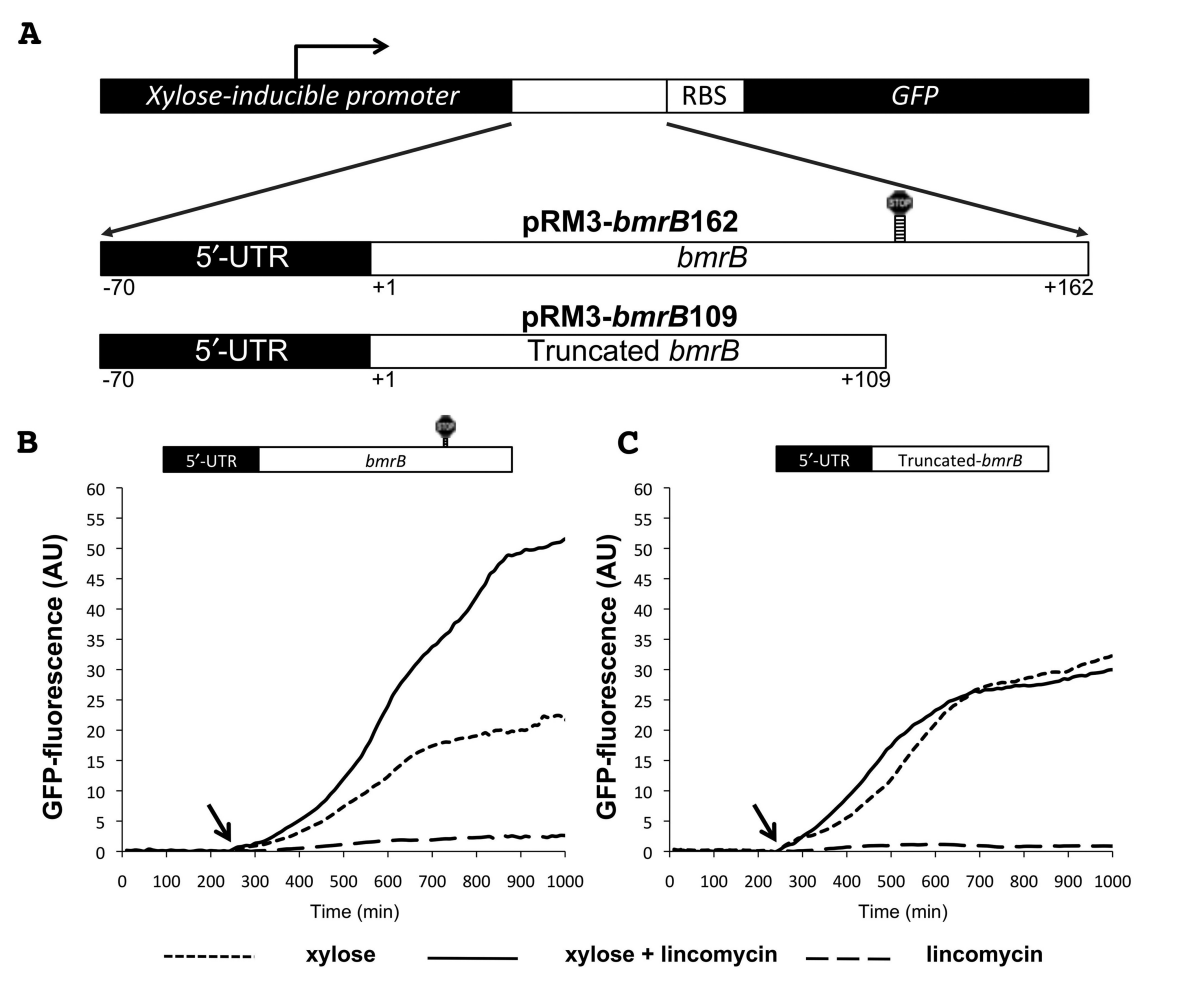
The 3′ end of *bmrB* is required for lincomycin-induced *bmrCD* transcription. **A.** Schematic representation of a part of plasmids pRM3-*bmrB*162 and pRM3-*bmrB*109. pRM3-*bmrB*162 was constructed by fusing the complete sequence of *bmrB* and its 5′-UTR to the *gfp* gene. pRM3-*bmrB*109 was constructed by fusing a truncated version of *bmrB*, consisting of this gene's first 109 nucleotides to *gfp.* Accordingly, pRM3-*bmrB*109 lacks the predicted terminator within *bmrB* and part of the predicted anti-terminator. Panels **B** and **C** show the GFP fluorescence of *B. subtilis* carrying pRM3-*bmrB*162 (**B**) or pRM3-*bmrB*109 (**C**) in response to xylose, lincomycin (0.75 μg/ml), or a combination of xylose and lincomycin added at the time points indicated by arrows. Xylose-induced GFP fluorescence, given in AU, was used as measure for the level of *gfp* expression.

As demonstrated in our LCA experiments, the promoter controlling the expression of the *bmrBCD* operon itself proved to be unresponsive toward the tested antibiotics. Therefore, we next determined whether the antibiotic-induced expression of *bmrCD* could be regulated via the putative intrinsic terminator located within the *bmrB* sequence. To test this hypothesis, we constructed two transcriptional GFP fusions consisting of either a complete (1–162 bp) or a truncated version (1–109 bp) of the *bmrB-*coding sequence, the latter lacking the terminator region. In addition, the 5′ UTR, assigned according to the *B. subtilis* Expression Data Browser (17), was included in the design (Figure 5A and Supplementary Figure S1). The two resulting transcriptional GFP fusions were cloned into pRM3, a plasmid for xylose-inducible gene expression in *B. subtilis*. Specifically, the resulting plasmid pRM3-*bmrB*162 carried the complete *bmrB* sequence, while pRM3-*bmrB*109 carried the truncated version of *bmrB* (Figure 5A). Strains expressing these transcriptional fusions upon induction with 0.1% xylose (final concentration; w/v) were monitored for growth and GFP production in the presence or absence of 0.75 μg/ml lincomycin.

When pRM3-*bmrB*162 was induced with xylose, GFP fluorescence increased gradually to a maximum of ∼22 AU (Figure 5B). The fact that we observed expression of GFP was an indication that the *gfp* gene was transcribed and translated. In addition, this observation demonstrated that although the *bmrB* terminator is present in pRM3-*bmrB*162, it was not able to prevent transcription of *gfp* completely. By co-induction with xylose and lincomycin, the GFP fluorescence from pRM3-*bmrB*162 was more than doubled, reaching ∼53 AU. Lincomycin alone did not induce expression of GFP. This indicates that lincomycin has no influence on transcription initiation, but rather enhances the transcriptional elongation of *bmrB*.

**Figure 6.**
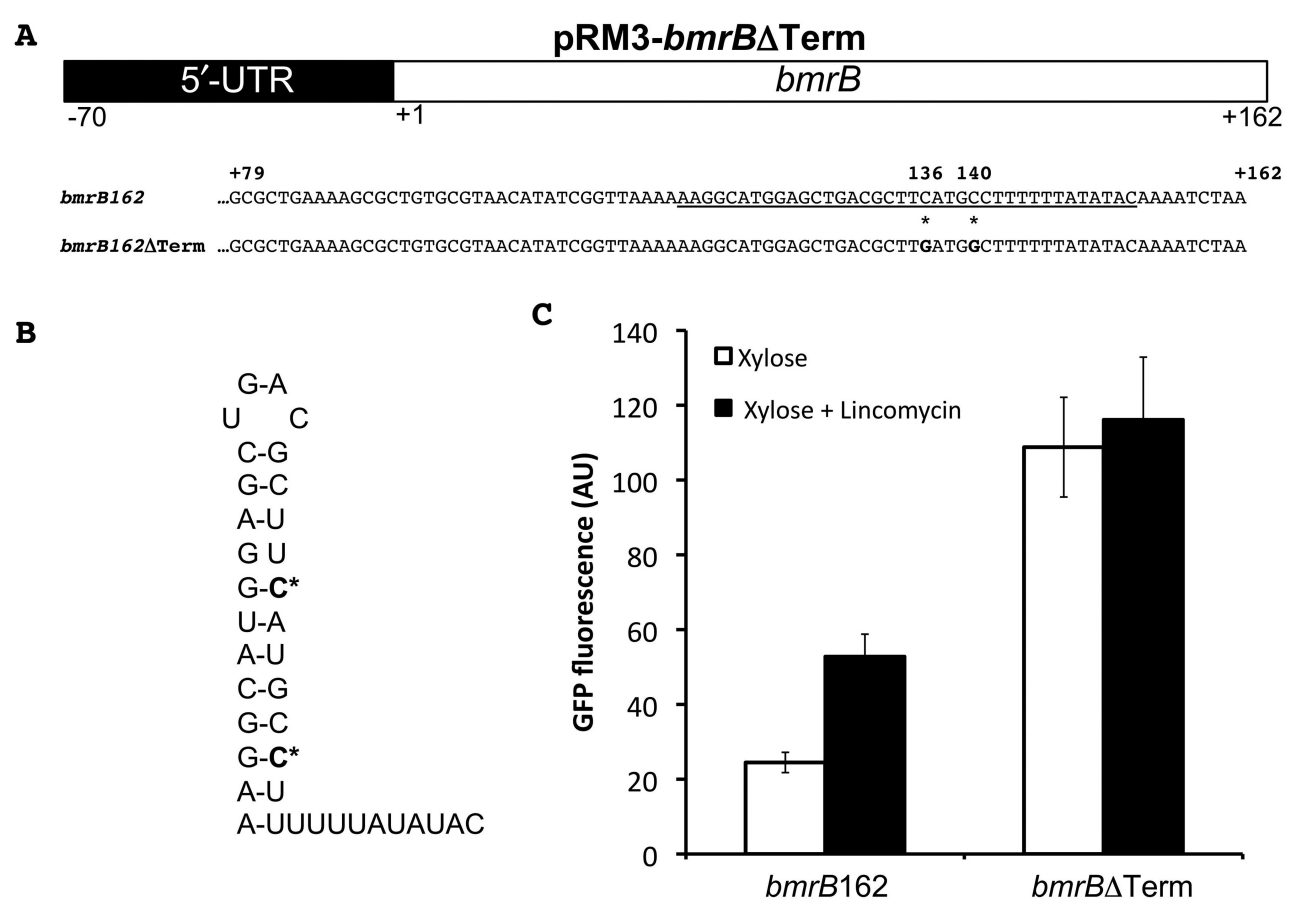
The terminator in *bmrB* is required for lincomycin-induced *bmrCD* transcription. **A.** Schematic representation of a part of plasmid pRM3-*bmrB*ΔTerm. Nucleotides 79–162 of the intact *bmrB* gene and its *bmrB*ΔTerm derivative are shown; C to G point mutations at positions 136 and 140 are marked with an asterisk (*). The predicted terminator sequence is underscored. **B.** Predicted structure of the terminator sequence with the position of the C to G point mutations marked in bold and with an asterisk (*). **C.** Maximum GFP expression levels measured for *B. subtilis* strains carrying pRM3-*bmrB*162 (marked *bmrB*162) or pRM3-*bmrB*ΔTerm (marked *bmrB*ΔTerm) upon induction with xylose (white bars) or xylose and 0.75 μg/ml lincomcyin (black bars).

To assess whether the *bmrB* terminator was involved in the lincomycin-mediated control, we used pRM3-*bmrB*109. In the strain carrying this plasmid, xylose-induced GFP levels reached a maximum of ∼33 AU (Figure 5C), which is a slightly higher level than that observed for the strain carrying pRM3-*bmrB*162 (Figure 5B). However, by removing the terminator sequence, the lincomycin-mediated control of the xylose-induced GFP expression was completely lost. *B. subtilis* pRM3-*bmrB*109 induced with xylose and lincomycin, produced fluorescence levels of ∼31 AU, which were similar to those of the xylose-induced strain without lincomycin (Figure 5C). The latter shows that the region containing the *bmrB* terminator sequence is essential for the lincomycin-mediated control observed for pRM3-*bmrB*162. To verify the role of the terminator structure in controlling the lincomycin-induced transcription of *bmrCD*, we constructed pRM3-*bmrB*ΔTerm (Figure 6A). In this reporter construct the formation of the terminator is precluded, according to a RibEx: Riboswitch Explorer (18) prediction, due to the introduction of two C to G point mutations at positions 136 and 140 (Figure 6A and B). In *B. subtilis* pRM3-*bmrB*ΔTerm, the induction with xylose alone was already sufficient to reach maximum GFP levels of ∼109 AU, which corresponded to a ∼5-fold increase compared to xylose-induced expression of the *bmrB*162-*gfp* fusion (Figure 6C). Importantly, lincomycin-mediated control of GFP expression was completely absent in the strain carrying pRM3-*bmrB*ΔTerm. Induction with xylose and lincomycin resulted in an average GFP level of ∼115 AU, which was comparable to that observed in the xylose-induced cultures without lincomycin (Figure 6C). First, these observations imply that formation of the terminator structure is essential for lincomycin-mediated control of *bmrCD* expression. Second, the elevated GFP expression due to the two point mutations in the *bmrB*ΔTerm-*gfp* fusion indicates that the predicted terminator does indeed set a limit to the expression of *bmrCD*. Finally, the findings presented in Figure 6C show that lincomycin can significantly enhance GFP expression in strains containing the *bmrB*162*-gfp* fusion, but that this antibiotic cannot completely prevent the termination event, at least at the applied lincomycin concentration. It thus seems that antibiotic-induced transcription of *bmrCD* is at least partly controlled via the intrinsic terminator within *bmrB*. Noteworthy was the observed discrepancy between the GFP expression levels directed from pRM3-*bmrB*109 (Figure 5C) and pRM3-*bmrB*ΔTerm (Figure 6C). This observation suggests that the removal of 53 bp of the 3′end of *brmB*, including the terminator plus a part of the putative anti-terminator structure (Figures 1B and 5A), has a negative influence on the GFP expression levels. In this respect, the use of point mutations, as applied in pRM3-*bmrB*ΔTerm, represents a more commendable strategy.

### Transcription of *bmrCD* is controlled via a ribosome-mediated attenuation mechanism

With the aid of pRM3-*bmrB*109 and pRM3-*bmrB*ΔTerm, we demonstrated that the terminator within the coding region of *bmrB* is able to regulate the expression of the downstream *bmrCD* genes via a transcriptional attenuation mechanism, which seems responsive to several ribosome-targeted antibiotics. We therefore aimed to further characterize the molecular details of this antibiotic-mediated regulatory mechanism. Although their precise mechanism of action varies, all ribosome-targeted antibiotics interfere with protein synthesis. Since *bmrB* contains a 53 amino acid open reading frame (ORF), we wondered whether *bmrB* could encode a potential leader peptide involved in the regulation of transcriptional read-through via its translation.

To determine whether *bmrB* is translated and could thus function as a regulatory leader peptide, we constructed a translational BmrB-GFP fusion, using the pMUTIN-GFP chromosomal integration plasmid (20). In this case, *gfp* was fused in-frame with the predicted *bmrB*-coding sequence at the *bmrB* locus (Supplementary Figure S4A). However, cells containing this construct showed no detectable GFP fluorescence. To test whether this was due to low-level expression of BmrB-GFP from the single-copy gene fusion on the chromosome, the in-frame *bmrB-gfp* fusion was PCR-amplified and cloned in plasmid pRM3. Upon induction with xylose, cells containing the resulting construct (pRM3-*bmrB-gfp*Inframe) were fluorescent, which showed that the *bmrB* open reading frame is translated and, therefore, has the intrinsic potential to act as a regulatory leader peptide (Supplementary Figure S4).

**Figure 7.**
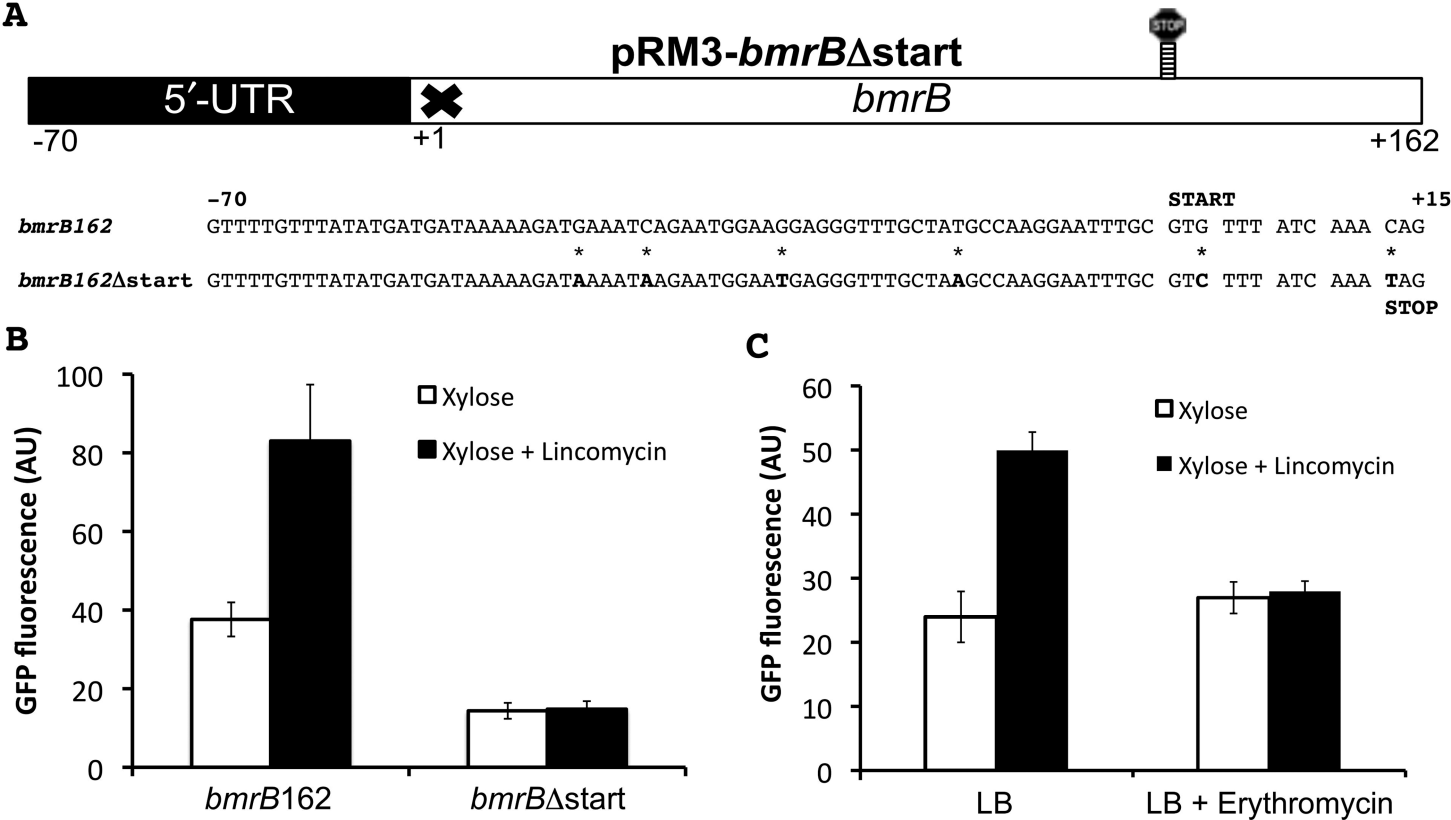
Translation of *bmrB* is essential for efficient and lincomycin-induced transcription of *bmrCD*. **A.** Schematic representation of plasmid pRM3-*bmrB*Δstart with the sequences of the first 15 nucleotides of *bmrB* and *bmrB*Δstart, and 70 nucleotides of the 5′-UTR. The mutations that were introduced to preclude translation of *bmrB* are marked with an asterisk (*). **B.** Maximum GFP expression values (AU) obtained from *B. subtilis* carrying pRM3-*bmrB*162 or pRM3-*bmrB*Δstart in response to xylose induction, either with or without lincomycin (0.75 μg/ml). **C.** ErmC-mediated methylation of the lincomycin-binding site on the ribosome prevents lincomycin-induced expression of *bmrB-gfp*. *B. subtilis* cells carrying pRM3-*bmrB*162 were pre-cultured in medium with or without erythromycin (0.5 μg/ml) and maximum GFP expression values were measured upon xylose induction, either with or without lincomycin (0.75 μg/ml). Culturing of cells in the presence of erythromycin induces expression of the *ermC* gene located on pRM3.

To test the hypothesis that *bmrB* could function as a leader peptide, we constructed the transcriptional pRM3-*bmrB*Δstart-*gfp* fusion in which we prevented the translation of *bmrB*. This was done by mutating the annotated GTG start codon to GTC, the insertion of a TAG stop codon downstream of the mutated start codon, and four point mutations in the 5′-UTR that either remove alternative start codons or potential RBSs (Figure 7A). As shown in Figure 7B, cells carrying the resulting plasmid pRM3-*bmrB*Δstart produced ∼15 AU of GFP upon xylose induction. This was substantially lower than the GFP expression observed for xylose-induced cells carrying pRM3-*bmrB*162 (Figure 7B), which suggested that impaired translation of *bmrB* increases the efficiency of the transcriptional terminator within the *bmrB* coding sequence. More importantly, expression of the *bmrB*Δstart-*gfp* fusion was not inducible with lincomycin (Figure 7B). Together, these results imply that translation of *bmrB* is required for efficient and lincomycin-inducible transcription of *bmrCD*, supporting our hypothesis that BmrB could function as a sensory leader peptide in a transcriptional attenuation mechanism.

In a leader peptide-based transcriptional attenuation mechanism for *bmrCD*, the ribosome could conceivably act as a sensory component for the presence of antibiotics which, in turn, would affect the rate of translation of *bmrB*. If so, transcription downstream of the *bmrB* terminator would depend on the effect that lincomycin exerts on the ribosome. To obtain experimental evidence for an involvement of the ribosome–lincomycin interaction in the translation-mediated transcriptional read-through of the *bmrB* terminator, we took advantage of the *ermC* selection marker on pRM3. The encoded erythromycin ribosomal methylase provides resistance to macrolides, lincosamides and streptogramins by methylating a single adenine residue of the 23S rRNA (the rRNA component of the 50S subunit), thereby preventing binding of the antibiotic compound. While *ermC* provides cross-resistance to several ribosome-targeted antibiotics, including lincomycin, expression of *ermC* can only be induced by erythromycin (21). To induce methylation of the ribosomes, the strain containing pRM3-*bmrB*162 was cultured in LB with a subinhibitory concentration of 0.5 μg/ml erythromycin. As a result of this, the lincomycin-mediated control on the expression of GFP was completely lost. Specifically, the induction with xylose alone resulted in GFP levels similar to those of cells grown in LB with xylose and lincomycin (∼25 AU) (Figure 7C). The fact that ribosome methylation prevented the antibiotic-controlled regulation of *bmrCD* underpins the importance of the antibiotic–ribosome interaction, which is apparently required for read-through transcription beyond the *bmrB* terminator-like sequences.

Taken together, these observations show that the antibiotic-mediated regulation of transcriptional termination occurs during the translation of *bmrB.* The BmrB peptide would then serve as a regulatory leader peptide. In this system, the ribosomes translating the BmrB leader peptide would act as a sensor for the presence of ribosome-targeted antibiotics. By inhibiting protein synthesis, these antibiotics will interfere with the efficient translation of *bmrB*. In turn, this would promote the formation of the anti-termination structure, thereby stimulating the transcription of the downstream *bmrCD* genes.

### Rare codons in *bmrB* affect the transcriptional attenuation decision

**Figure 8.**
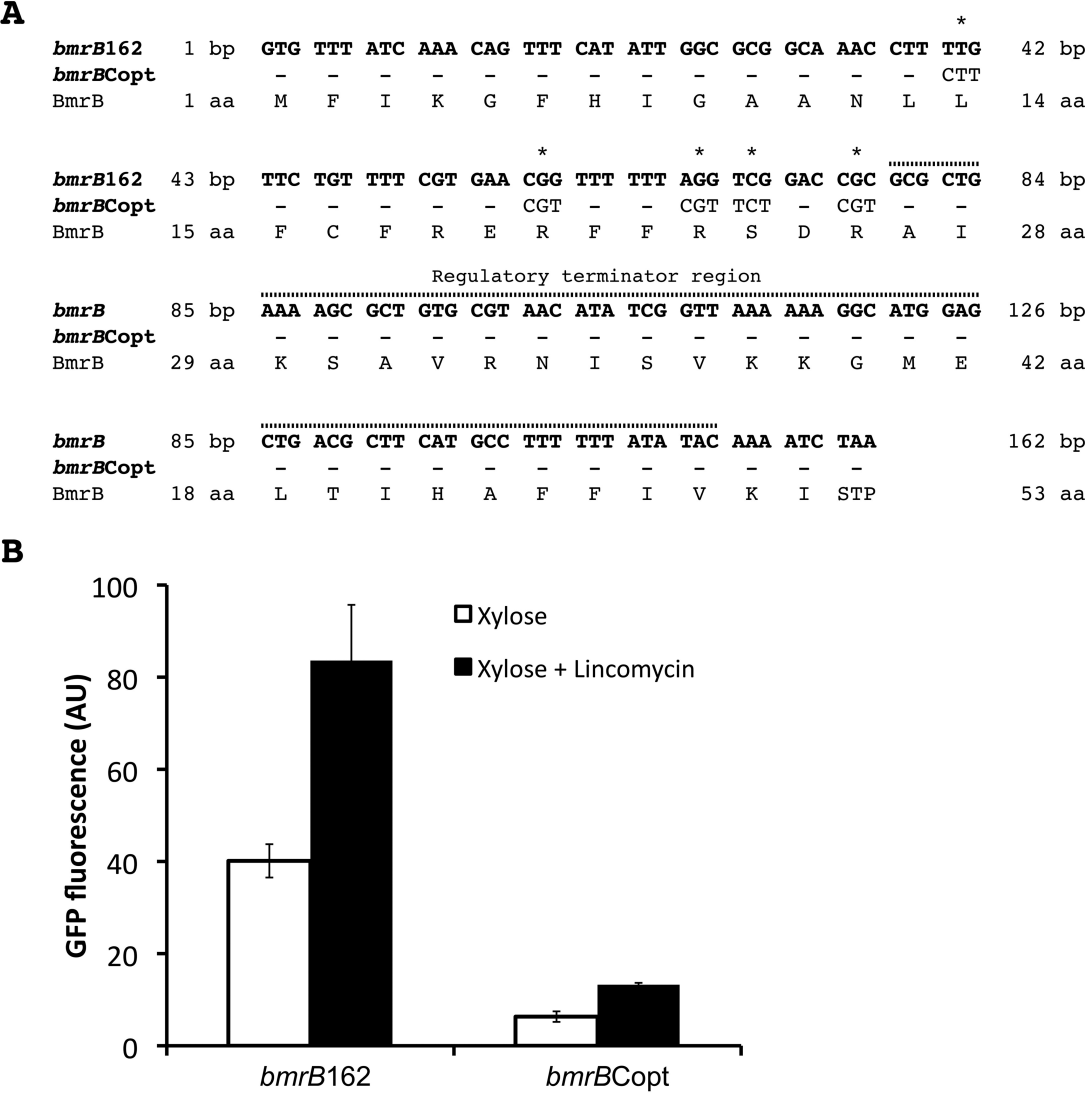
Codon optimization in *bmrB* leads to reduced transcription of *bmrCD*. **A.** Sequences of the *bmrB* (marked *bmrB*162) and *bmrB*Copt genes. The five rare codons that were mutated in *bmrB*Copt are marked with an asterisk (*). **B.** Maximum GFP expression values (AU) obtained from *B. subtilis* carrying pRM3-*bmrB*162 or pRM3-*bmrB*Copt in response to xylose induction, either with or without lincomycin (0.75 μg/ml).

The importance of the translation of *bmrB* for controlling the transcription of *bmrCD* led us to ask the question whether the codon composition of *bmrB* could be important for its function as a regulatory leader peptide. This question was relevant since we have identified five rare codons within the *bmrB* coding sequence upstream of the regulatory terminator region (Figure 8A). The rare codons were manually assigned based on the previously determined codon usage in *B. subtilis* (22) and this was verified using the online codon optimization tool JCat (http://www.jcat.de/) (23). Rare codons are known to slow down the speed of translation (24–27), and, therefore, the presence of these rare codons within *bmrB* could affect the transcriptional termination decision. To investigate this idea, a codon-optimized *bmrB* sequence was designed using the JCat tool and subsequently synthesized. This codon-optimized *bmrB* sequence was transcriptionally fused to *gfp* and cloned into pRM3, which resulted in plasmid pRM3-*bmrB*Copt (Figure 8A). Next, the GFP expression by cells containing pRM3-*bmrB*Copt was compared to that of cells containing pRM3-*bmrB*162. Interestingly, the xylose-induced GFP expression from pRM3-*bmrB*Copt reached a level of ∼6.3 AU, which was much lower than the xylose-induced GFP expression of ∼40 AU from pRM3-*bmrB*162 (Figure 8B). Furthermore, co-induction of the *bmrB*Copt-*gfp* fusion with xylose and lincomycin doubled the GFP expression levels to ∼13 AU, and this 2-fold increase was comparable to the increase observed for the *bmrB*162-*gfp* fusion induced with xylose and lincomycin, which reached ∼82 AU. Thus, although the lincomycin-mediated regulation was not affected by the codon optimization, the total GFP expression levels directed from pRM3-*bmrB*Copt were considerably lower compared to those directed from pRM3-*bmrB*162. This suggests that the codon-optimized *bmrB* sequence results in more efficient termination of the *bmrB*-*gfp* transcript, without affecting the regulatory capacity of the terminator region. It therefore seems that the rare codons in *bmrB* slow down the rate by which the corresponding mRNA is translated, and this might provide sufficient time for the predicted anti-terminator to form. In contrast, codon optimization of the *bmrB* sequence increases the rate of translation, allowing less time for the anti-terminator structure to form, and thereby favors transcriptional termination. The latter will result in the lower GFP levels as documented in Figure 8B. Altogether, our observations suggest that the rare codons in *bmrB* do not play a major role in the lincomycin-mediated control, because the regulatory capacity of the codon optimized *bmrB* sequence remained apparently unaffected. Instead these rare codons seem to enhance the basal level of transcriptional read-through. In addition, these results emphasize the importance of the rate of translation in the regulation of read-through transcription beyond *bmrB*.

## DISCUSSION

In the present study, we have employed promoter-GFP fusions to monitor the expression dynamics of the genes encoding the MDR-ABC transporter BmrCD upon exposure to antibiotics. This real-time approach revealed that the antibiotic-induced expression profile of *bmrCD* is constrained to the transition and stationary growth phases due to a tight control by the transition state regulator AbrB. In addition, we have demonstrated that antibiotic-induced expression of *bmrCD* is regulated via a ribosome-mediated transcription termination mechanism, a regulatory mechanism that has not been previously described for MDR-ABC transporters.

Our study shows that the promoter controlling transcription of the *bmrBCD* operon is regulated via the transcriptional regulator AbrB, which is known to repress stationary phase-specific genes during exponential growth of *B. subtilis* (28), and that this regulation determines the timing of antibiotic-induced *bmrCD* expression. Nevertheless, although the AbrB-regulated promoter upstream of *bmrB* controls the growth phase-dependent expression of *bmrCD*, this promoter is unresponsive to the antibiotics that induce *bmrCD* expression. By constructing several different transcriptional-fusions, we have now demonstrated that the antibiotic-induced expression of these two MDR-transporter genes is regulated via the transcriptional terminator located within the coding sequence of *bmrB.* In the absence of antibiotics, such as lincomycin, transcription of the *bmrBCD* operon will be terminated at this terminator site, thereby minimizing expression of *bmrCD.* In contrast, when the cells are challenged with translational inhibitors, such as lincomycin, erythromycin or chloramphenicol, transcriptional termination is prevented and the *bmrCD* transporter genes are transcribed. Moreover, our study shows that translation of *bmrB* is essential for the antibiotic-controlled transcription of *bmrCD*. From these observations we conclude that BmrB acts as a regulatory leader peptide, and that the extent of *bmrCD* transcription is related to the efficiency of *bmrB* translation. Intriguingly, kanamycin and gentamycin, both targeting the 30S ribosomal subunit, were unable to induce the 5′*bmrC-gfp* fusion, whereas chloramphenicol, erythromycin and lincomycin, which target the 50S subunit, did induce the 5′*bmrC-gfp* fusion. This difference between antibiotics that target the 50S and 30S ribosomal subunits could relate to their exact binding sites on the ribosome and/or mechanisms of action in relation to *bmrB* translation, but this is presently highly speculative and further research is needed to explain this observation.

**Figure 9.**
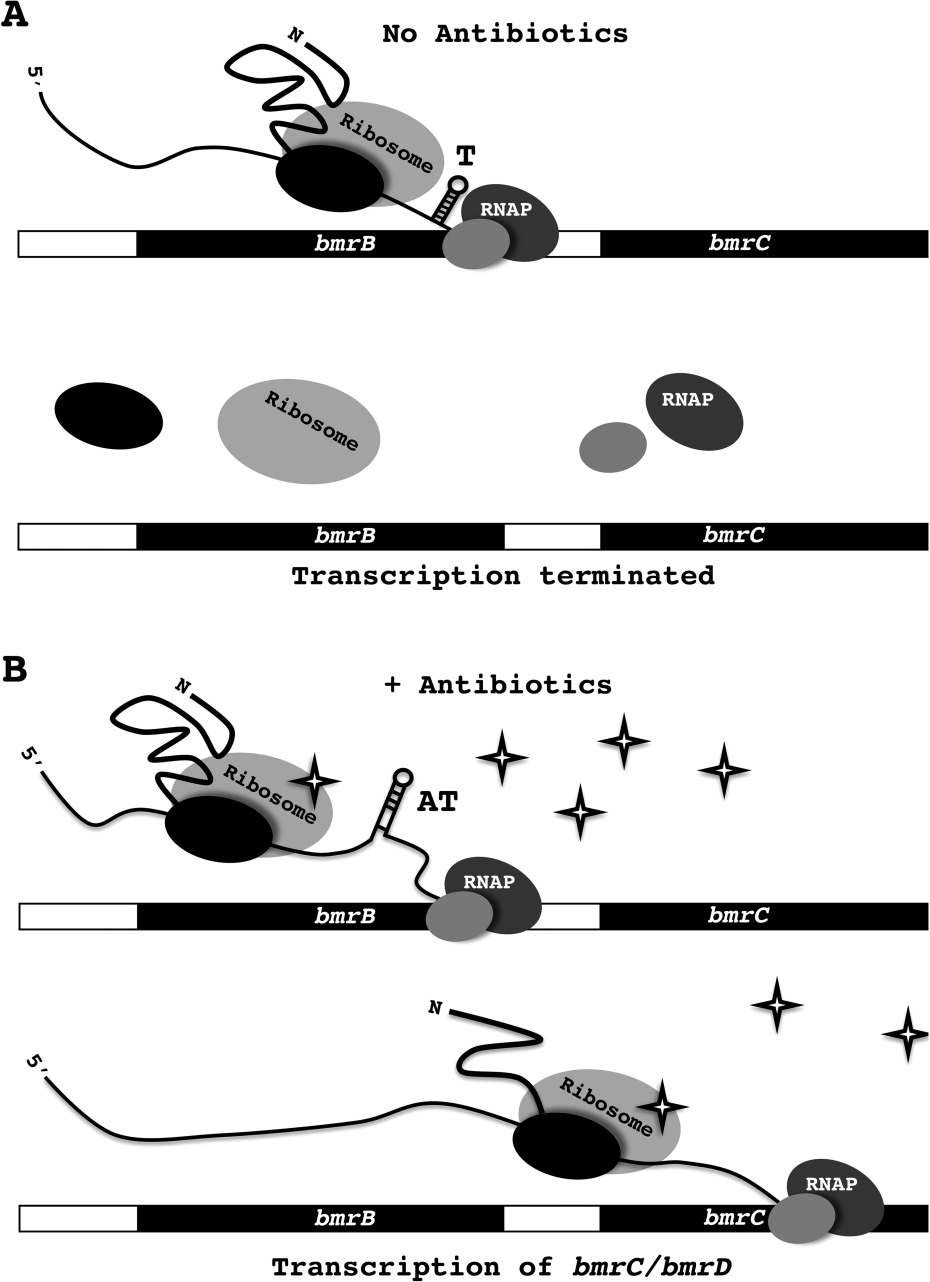
Model for antibiotic-induced *bmrCD* expression. Due to transcriptional and translational coupling, the RNA polymerase transcribing the *bmrB* gene is closely followed by the respective ribosome-mRNA-BmrB nascent chain complex. **A.** In the absence of ribosome-targeted antibiotics, the ribosome can translate the *bmrB* transcript without impediments. When *bmrB* is efficiently translated the formation of an anti-terminator structure, which is energetically less favorable than the terminator structure (Figure 1C), is prevented. Consequently, this will result in formation of the terminator structure (T), which will, by destabilizing the transcriptional elongation complex, result in transcriptional termination. **B.** The presence of antibiotics, such as lincomycin (indicated as stars), which target the bacterial ribosome, will reduce the translation rate of *bmrB*. This will allow formation of the anti-terminator (AT) structure and, thereby, preclude formation of the transcriptional terminator. In turn, this will allow the RNA polymerase to continue transcription of *bmrCD*.

While we have identified the first leader peptide-based transcriptional control system for the expression of an MDR transporter, it should be noted that leader peptide-based regulatory mechanisms have previously been reported to control various other processes in bacteria. These mechanisms rely on the fact that bacterial transcription and translation are coupled. The best-known example of leader peptide control involving transcriptional termination is the operon responsible for tryptophan synthesis in *E. coli* (29,30). In this case, the Trp leader peptide contains two adjacent tryptophan residues involved in the sensing of tryptophan shortage, which will result in stalling of the translating ribosome at the adjacent Trp codons (31). This ribosome stalling promotes the formation of an anti-terminator structure and thereby prevents premature termination of the transcript (32). The regulation of the *B. subtilis bmrBCD* operon, as described here, differs from that of the *E. coli trp* operon in that ribosome stalling is caused by the inhibitory effect of the ribosome-targeted antibiotics on protein synthesis instead of a tryptophan shortage. However, as described for the *trp* operon, it seems likely that the ribosome translational stalling will allow structural organization of the anti-terminator in *bmrB*, thereby promoting the transcription of *bmrCD* as schematically represented in Figure 9. In this way, the ribosome plays a regulatory role in transcriptional regulation by acting as a sensor for compounds like lincomycin that hinder efficient translation. Indeed, our results show that the interaction between the ribosome and the antibiotic is crucial for the anti-termination process, especially since the lincomycin-induced *bmrB* transcription was completely lost upon methylation of the ribosome by ErmC (Figure 7C). The function of the predicted anti-anti-terminator (Figure 1C) within our proposed transcriptional attenuation model remains unclear. If functional, it is conceivable that formation of the anti-anti-terminator structure promotes the formation of the terminator, and thus, acts as an inhibiting factor for *bmrCD* transcription.

Transcriptional termination has previously been reported as a regulatory mechanism in the expression of several resistance genes, including the *B. subtilis vmlR* ABC transporter gene, which is involved in resistance to lincomycin and virginiamycin M (33,34). These ribosome-targeted antibiotics also promote transcriptional read-through and thus expression of *vmlR* (34). However, the 5′UTR of *vmlR* lacks an ORF, indicating that the expression of this gene is not regulated via ribosome-mediated transcriptional termination. In addition, *vmlR* lacks the typical anti-terminator structure that is normally required for such transcriptional attenuation mechanisms, and the precise mechanism regulating *vmlR* expression remains to be determined (34). Furthermore, expression of *ermC*, encoding the erythromycin ribosomal methylase C, is regulated via another class of leader peptide-based systems. Under non-inducing conditions efficient translation of the leader peptide of *ermC* promotes formation of a stem-loop structure that protects the RBS so that translation of *ermC* is prevented (21). The presence of erythromycin causes stalling of the ribosome that is translating the *ermC* leader peptide, giving time for the formation of an alternative stem-loop structure (35). As a result, the RBS becomes accessible, and ribosomes can efficiently translate *ermC* (36). The regulatory control mechanism of *ermC* encourages the theory that translational leader peptides can act as efficient sensors for ribosome-targeted antibiotics. This is further supported by the translation-mediated attenuation mechanism controlling transcription of *cat-86*, a plasmid-borne chloramphenicol resistance gene in *B. subtilis*. Expression of *cat-86* is induced when the ribosome, while translating the leader peptide, is stalled by chloramphenicol (37). Despite some overlap with the regulatory mechanisms of *vmlR*, *ermC* and *cat-86*, the *bmrCD* genes are, to the best of our knowledge, the first MDR-ABC transporter genes to be regulated via ribosome-mediated transcriptional attenuation. In fact, the termination system controlling the expression of *bmrCD* is the first documented transcriptional attenuation mechanism encoded in the *B. subtilis* genome that is controlled by a leader peptide. However, it should be noted that other transcriptional attenuation systems have previously been characterized in *B. subtilis*. One example is the tryptophan synthesis operon, which is dependent on an RNA-binding protein to control expression of downstream genes (38). Additionally, *B. subtilis* has several transcriptional systems that are controlled by riboswitches (39). These RNA elements control transcription of downstream genes by changing their structure in response to temperature shifts, or direct interaction with cellular components (e.g. tRNAs) or small molecules (e.g. amino acids, metals) (40).

The biological relevance for the observed strictly growth phase-dependent *bmrCD* expression is presently elusive. Fast and efficient detoxification of the cellular interior is essential when bacteria are exposed to antibiotics. Therefore, most drug efflux systems show an immediate transcriptional response toward the toxic compounds they expel. However, as a soil bacterium, *B. subtilis* can be confronted with a large variety of different antimicrobial compounds and this is probably the reason why it possesses a multitude of efflux pumps (3). Consequently, it seems most likely that there is overlap in the substrate specificities of the different MDR transporters of *B. subtilis*. The transcriptional response of *bmrCD* is strongest upon exposure to lincomycin, an antibiotic produced by *Streptomyces lincolnensis*, which is also a soil-dwelling bacterium (41). Interestingly, the lincomcyin-induced expression of *vmlR* occurs during the early- and mid-exponential growth phases and declines in the late-exponential growth phase (34). It thus partially overlaps with the lincomycin-induced *bmrCD* expression during the late-exponential and stationary growth stages. In addition, *lmrB*, encoding an additional lincomycin exporter, follows a similar expression profile as *vmlR* (42,43). Since both *vmlR* and *lmrB* seem to be transcribed predominantly in the exponential growth phase, it appears that expression of *bmrCD* would be redundant during this point in growth and more effective during later growth stages. An alternative explanation for the strictly growth phase-dependent expression profile of *bmrCD* is a potential role of the respective transporter in sporulation. Transcript levels of *bmrCD* increase during sporulation (17), and the overexpression of *bmrCD* was shown to significantly reduce spore formation in a *kinB* mutant *B. subtilis* strain (42). Kumano *et al.* subsequently demonstrated that BmrD interacts with KinA, one of the key regulators of the sporulation process, and they postulated that this interaction might inhibit the activation of KinA by trapping it to the inner surface of the cytoplasmic membrane.

In conclusion, the regulatory mechanism controlling the transcription of the *bmrCD* genes as uncovered in the present study points toward a role of BmrCD in antibiotic efflux during the post-exponential stages of growth. This mechanism is apparently tailored for the optimal extrusion of ribosome-targeted antibiotics and represents the first ribosome-mediated transcriptional attenuation system described for an MDR-ABC transporter.

## SUPPLEMENTARY DATA

Supplementary Data are available at NAR Online.

SUPPLEMENTARY DATA
