# Supplementary material for: The multidrug ABC transporter BmrC/BmrD of Bacillus subtilis is regulated via a ribosome-mediated transcriptional attenuation mechanism
Source: Nucleic Acids Res. 2014 Sep 12;42(18):11393–407. doi: 10.1093/nar/gku832 (PMC4191407; doi:10.1093/nar/gku832)
Supplement: SUPPLEMENTARY DATA [file supp_42_18_11393__index.html]

The multidrug ABC transporter BmrC/BmrD of Bacillus subtilis is regulated via a ribosome-mediated transcriptional attenuation mechanism — SUPPLEMENTARY DATA 

# The multidrug ABC transporter BmrC*/*BmrD of *Bacillus subtilis* is regulated via a ribosome-mediated transcriptional attenuation mechanism

## SUPPLEMENTARY DATA

**Files in this Data Supplement:**

- SUPPLEMENTARY DATA
