# Supplementary material for: The multidrug ABC transporter BmrC/BmrD of Bacillus subtilis is regulated via a ribosome-mediated transcriptional attenuation mechanism
Source: Nucleic Acids Res. 2014 Sep 12;42(18):11393–407. doi: 10.1093/nar/gku832 (PMC4191407; doi:10.1093/nar/gku832)
Supplement: SUPPLEMENTARY DATA [file supp_gku832_nar-03438-v-2013-File011.pdf]

Supplementary Figures and Tables

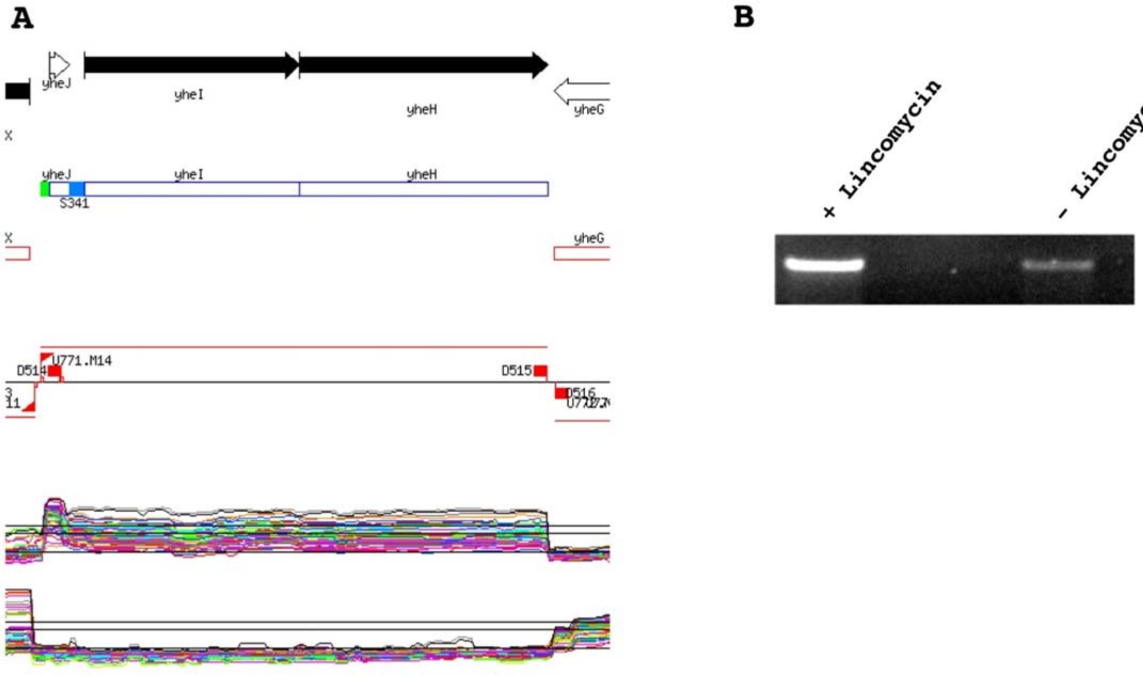

Supplementary Figure S1. The *bmrBCD* operon.
A. Snapshot of the *B. subtilis* Expression Data Browser (http://genome.jouy.inra.fr/cgi-bin/seb/viewsection.py?position=1045117), where *bmrB* (*yheJ*) and *bmrC*/*bmrD* (*yheI/yheH*) are annotated as a tricistronic operon (17). **B**. RT-PCR using RNA prepared from *B. subtilis* cultured with or without 0.5 µg/mL lincomycin. qPCR primers *bmrB*-forward and *bmrC*-reverse were used to confirm the *bmrBCD* operon structure. The increased intensity of the band derived from RT-PCR analysis on cells grown in the presence of lincomycin (+ lincomycin) indicates increased levels of *bmrBCD* transcription compared to cells grown in the absence of lincomycin (- lincomycin).

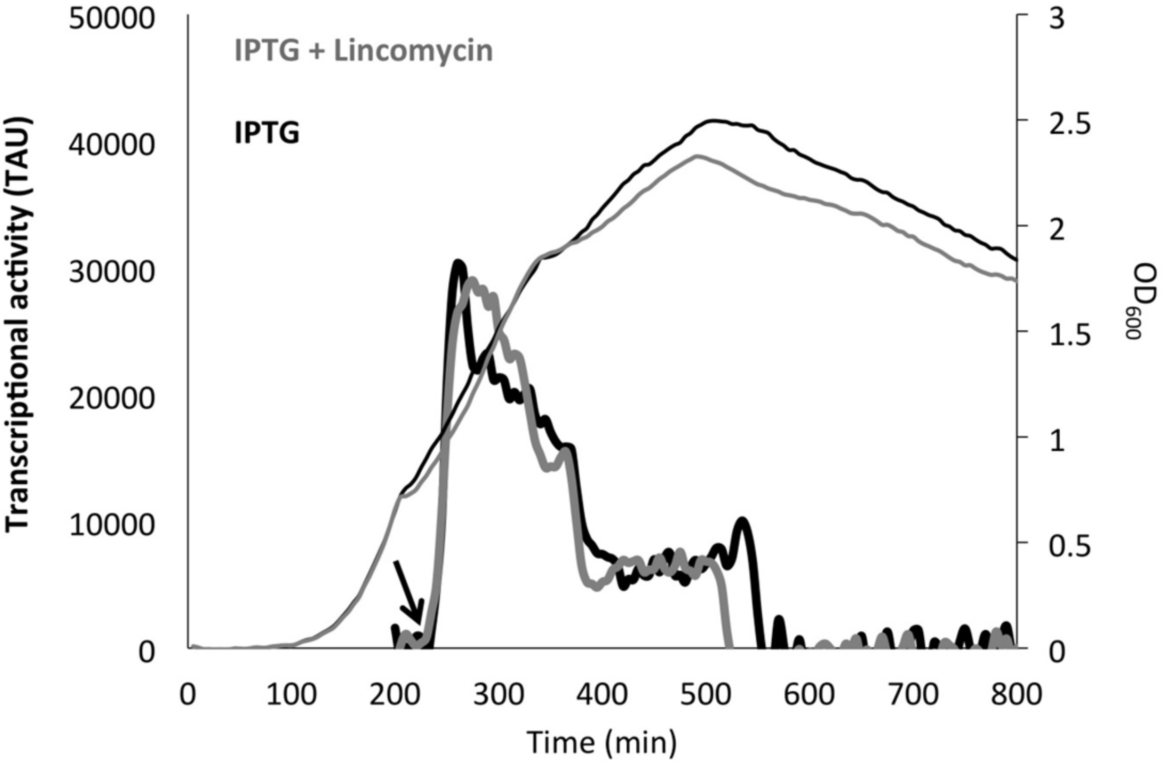

Supplementary Figure S2. Live cell array real-time analysis of IPTG-induced *gfp* transcription.

B. subtilis 168 *spac-gfp* was used to assess possible direct effects of lincomycin on GFP fluorescence**.** Growth (OD_{600}; thin lines) and *gfp* transcriptional activity (bold lines) were monitored in the presence of 0.1 mM IPTG either with 2.0 µg/mL lincomycin (grey line) or without lincomycin (black line). The arrow indicates the time point at which IPTG and lincomycin were added.

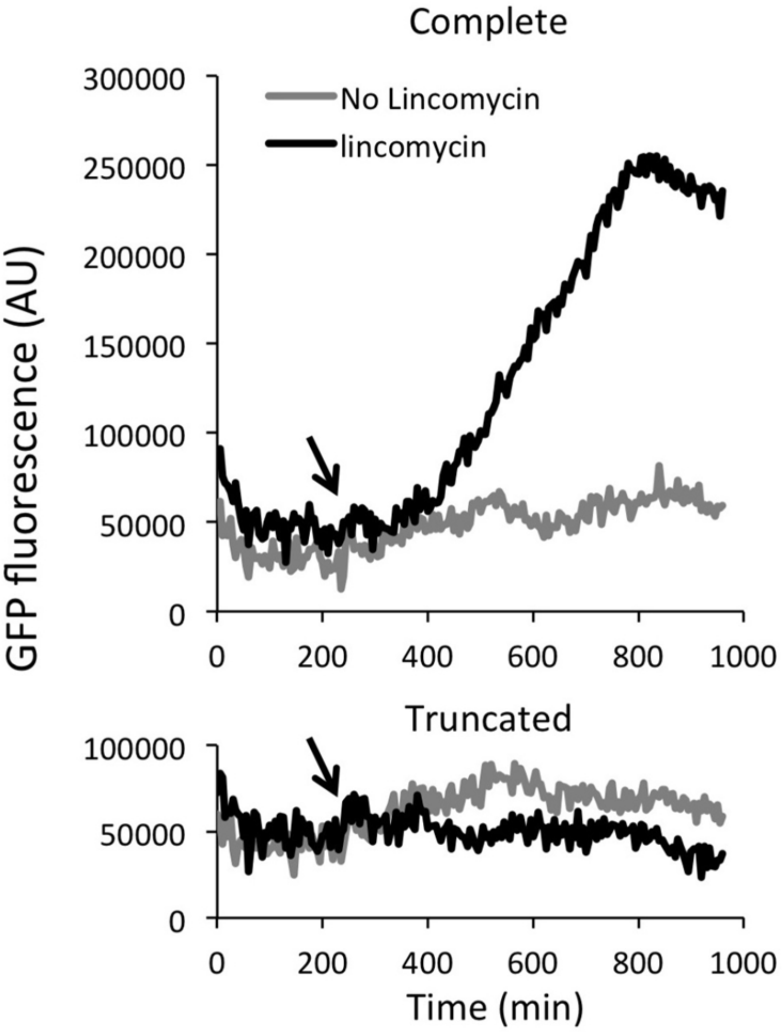

Supplementary Figure S3. Transcription of *bmrCD* is initiated from the *bmrBCD* promoter.
To determine whether transcription of the *bmrCD* genes is only directed from the *bmrBCD* promoter located in the upstream region of *bmrB*, two additional transcriptional 5’*bmrC-gfp* fusions were integrated in single copy in the *amyE* locus. These represented either the complete 5'*bmrC-gfp* fusion with the *bmrB* upstream region (see Figure 1), or a truncated version named 5’*bmrC*trunc-*gfp*, which lacks the sequences upstream of *bmrB* (including the *bmrBCD* promoter) up until the *bmrB* start codon. These complete or truncated versions of the 5'*bmrC*-GFP fusion were PCR-amplified from strain BSBII-*bmrC-gfp* using, respectively, the primer sets pRMC-5’*bmrC* forward and pRM3-*gfp* reverse, or pRMC-5’*bmrC*trunc forward and pRM3-*gfp* reverse. Next, the amplified fragments were cloned into pRMC and introduced via double cross-over recombination into the *amyE* locus. Cells containing the chromosomally integrated 5'*bmrC-gfp* or 5’*bmrC*trunc-*gfp* fusions were then analysed for GFP fluorescence. The upper panel shows the GFP fluorescence of cells containing the 5'*bmrC-gfp* fusion, which is induced upon addition of 0.2 µg/mL lincomycin (black line). The lower panel shows the GFP fluorescence of cells containing 5’*bmrC*trunc-*gfp* fusion, which does not increase upon addition of lincomycin. Arrows indicate the time point at which lincomycin was added. These results show that there is no additional promoter present in the region between the start codon of *bmrB* and *bmrC*, and that *bmrCD* transcription is initiated from a promoter upstream of *bmrB.*

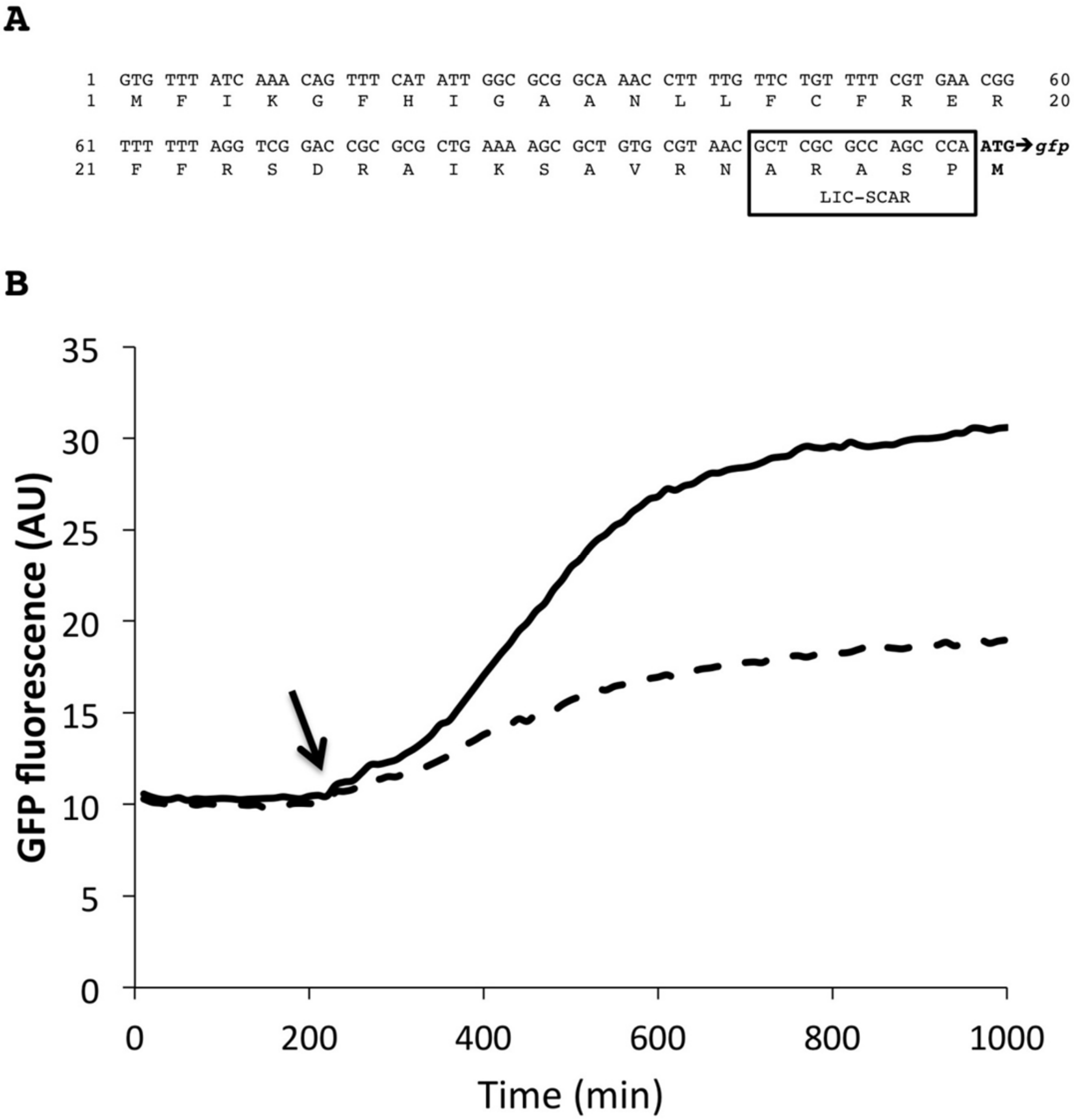

Supplementary Figure S4. Expression of an in-frame BmrB-GFP fusion protein.
A. Sequence of the *bmrB-gfp* in-frame fusion, including nucleotides 1-102 of *bmrB.* The ATG start codon of GFP is shown in bold. Due to the LIC procedure, five extra amino acid residues were included in the BmrB-GFP fusion, which are indicated by the box marked with LIC-SCAR. **B.** BmrB-GFP fluorescence (in arbitrary units) of cells carrying plasmid pRM3-*bmrB-gfp*Inframe grown either in the presence (solid line) or absence of xylose (dashed line).

Supplementary Table S1. Strains and plasmids

Strain	Description	Reference
B. subtilis 168 BSBII-5'*bmrB-gfp*	Strain carrying a chromosomally integrated copy of pBaSysBioII in which the ~600 bp region upsteam of *bmrB* was cloned. Accordingly, *gfp* transcription is directed from the 5' upstream region of *bmrB*.	This study
B. subtilis 168 BSBII-5'*bmrC-gfp*	Strain carrying a chromosomally integrated copy of pBaSysBioII in which the ~600 bp region upsteam of *bmrC* was cloned. Accordingly, *gfp* transcription is directed from the 5' upstream region of *bmrC*.	This study
B. subtilis 168 Pspac-*gfp*	Strain carrying a chromosomally integrated P*spac* promoter-*gfp* fusion. The P*spac*-directed transcription of *gfp* can be induced with IPTG; *amyE*::P*spac*-*gfp*mut2	(44)
B. subtilis 168 Δ*abrB*	*abrB* deletion strain	Laboratory collection
**Plasmids**		
pBaSysBioII	Chromosomal integration plasmid for construction of transcriptional *gfp*mut3 fusions, LIC compatible	(8)
pRM3	LIC compatible expression plasmid with a xylose-inducible promoter. Constructed by combining the pHB201 backbone and the *xylA* promoter region from pXTC.	(10, 11)<br>This study
pRM3-*bmrB*162	pRM3 carrying the complete *bmrB* gene (nucleotides 1-162) and its 5'-UTR (70 nucleotides)	This study
pRM3-*bmrB*109	pRM3 carrying a truncated copy of *bmrB* (nucleotides 1-109) and its 5'-UTR (70 nucleotides)	This study
pRM3-*bmrB*ΔTerm	pRM3-*bmrB*162 with mutated terminator sequence	This study
pRM3-*bmrB*Δstart	pRM3-*bmrB*162 with an untranslatable copy of *bmrB*	This study
pRM3-*bmrB*Copt	pRM3 including a codon-optimized *bmrB* sequence	This study
pMUTIN-GFP	Integrative LIC plasmid for construction of translational fusions *bla*R P*spac*-LIC-*gfpmut3*-*erm*R	(45)
pRM3-*bmrB-gfp*Inframe	pRM3 including an *in-frame* translational *bmrB-gfp* fusion	This study
pRMC	LIC-adapted derivative of the pXTC plasmid. Allows double cross-over recombination of *gfp* fusions in single copy into the *amyE* locus	(10). Laboratory collection
pRMC-5'*bmrC-gfp*	pRMC carrying the 5'*bmrC-gfp* fusion of BSBII-5'*bmrC-gfp* including the upstream region of *bmrB*	This study
pRMC-5'*bmrC*trunc-*gfp*	pRMC carrying the 5'*bmrC-gfp* fusion of BSBII-5'*bmrC-gfp*, but lacking the *bmrBCD* promoter region	This study

Supplementary Table S2. Primers

Primer set	Sequence
5'*bmrC* forward<br>5'*bmrC* reverse	CCGCGGGCTTTCCCAGCTGACGGTCTGATTGTCTTTCAT<br>GTTCCTCCTTCCCACCTCAGCCGCCTTCTATTTTT
5'*bmrB* forward<br>5'*bmrB* reverse	CCGCGGGCTTTCCCAGCGCTTTTTCAGCCTGTTTTGG<br>GTTCCTCCTTCCCACCGCAAACCCTCCTTCCATTCT
qPCR *recF* forward<br>qPCR *recF* reverse	GAGGGAAGAGTGATGAAGCA<br>CGACATACTGGCTGAGCTTT
qPCR *ssrA* forward<br>qPCR *ssrA* reverse	GAGAGGCGATCTCGTAAACA<br>TAGGCAATGTCAGGAAGAGC
qPCR *bmrB* forward<br>qPCR *bmrB* reverse	CGGCAAACCTTTTGTTCTG<br>GTCAGCTCCATGCCTTTTT
qPCR *bmrC* forward<br>qPCR *bmrC* reverse	ATTATCCCGCTTCCAGTCAT<br>TTCCAGCACTCTGTCATTCA
Prm3 construction:<br>phb20 forward + LICoverlap<br>phb201 reverse + pXTCoverlap<br>pXTC forward<br>pXTC reverse + LICsite	 **TGGCGCGCCAGCCCAAT**AGCCTGATACAGATTAAATCAGAACG<br>**CACCTGCTTTAGGAGCGGTT**TCTTCCGCTTCCTCGCTCA<br>AACCGCTCCTAAAGCAGGTG<br>**ATTGGGCTGGCGCGCCAGGAACCCT**TTTGGACCATTTGTCATTTCC
pRM3-*gfp* forward<br>pRM3-*gfp* reverse	GGAAGGAGGAACTACTATGCG<br>TTGGGCTGGCGCGAGCTTATTTGTATAGTTCATCCATGC
pRM3-*bmrB* forward	GGGTTCCTGGCGCGAGCGTTTTGTTTATATGATGATAAAAAG
pRM3-*bmrB*162 reverse	**TTTACGCATAGTAGTTCCTCCTTCC**TTAGATTTTGTATATAAAAAAGGC
pRM3-*bmrB*109 reverse	**TTTACGCATAGTAGTTCCTCCTTCC**CCGATATGTTACGCACAG
pRM3-*bmrB*ΔTerm forward<br>pRM3-*bmrB*ΔTerm reverse<br>pRM3-*bmrB*ΔTerm reverse with overlap (for joining PCR)	GATGGCTTTTTTATATACAAAATCTAAGGAAAG<br>AAGCGTCAGCTCCATGCC<br>**AGATTTTGTATATAAAAAAGCCATC**AAGCGTCAGCTCCATGCC
pRM3-*bmrB*Δstart reverse<br>pRM3-*bmrB*Δstart forward	GCTTTTCAGCGCGCGGTC<br>**TAGGTCGGACCGCGCGCTGAAAAGC**GCTGTGCGTAACATATCGGT
pRM3-*bmrB*Copt reverse<br>pRM3-*bmrB*Copt forward	GCTTTTCAGCGCACGGTCA<br>**TAGGTCGGACCGCGCGCTGAAAAGC**GCTGTGCGTAACATATCGGT
pRM3-*gfp* forward<br>pRM3-*gfp* reverse	GGAAGGAGGAACTACTATGCG<br>TTGGGCTGGCGCGAGCTTATTTGTATAGTTCATCCATGC

pRMC-5'*bmrC* forward	GGGTTCCTGGCGCGAGCTGACGGTCTGATTGTCTTTCAT
pRMC-5'*bmrC*trunc forward	GGGTTCCTGGCGCGAGCGTGTTTATCAAACAGTTTCATATTG
pMUTIN-*bmrB*-forward<br>pMUTIN-*bmrB*-reverse	GGGTTCCTGGCGCGAGCGCTTTTTCAGCCTGTTTTGGT<br>TTGGGCTGGCGCGAGCGTTACGCACAGCGCTTTTCA

Underlined are the 5'overhangs that were used for ligation-independent cloning
Depicted in bold are the 5'overhangs required for overlap PCR

Supplementary References

44. Piersma,S., Denham,E.L., Drulhe,S., Tonk,R.H., Schwikowski,B. and van Dijl,J.M. (2013) TLM-quant: An open-source pipeline for visualization and quantification of gene expression heterogeneity in growing microbial cells. *PLoS One*, **8,** e68696.

45. Doherty,G.P., Fogg,M.J., Wilkinson,A.J. and Lewis,P.J. (2010) Small subunits of RNA polymerase: Localization, levels and implications for core enzyme composition. *Microbiology*, **156,** 3532-3543.
